# Elucidating the role of shikimate dehydrogenase in controlling the production of anthocyanins and hydrolysable tannins in the outer peels of pomegranate

**DOI:** 10.1186/s12870-019-2042-1

**Published:** 2019-11-06

**Authors:** Rida Habashi, Yael Hacham, Rohit Dhakarey, Ifat Matityahu, Doron Holland, Li Tian, Rachel Amir

**Affiliations:** 1 MIGAL – Galilee Technology Center, 12100 Kiryat Shmona, Israel; 2 Tel-Hai College, 11016 Upper Galilee, Israel; 3 Newe Ya’ar Research Center, Agricultural Research Organization, 30095 Ramat Yishay, Israel; 4 Department of Plant Sciences, University of California Davis, Davis, California USA

**Keywords:** Anthocyanins, Hydrolysable tannins, Osmotic stress, Outer peel, Peel-tissue culture, Pomegranate, Shikimate dehydrogenase

## Abstract

**Background:**

The outer peels of pomegranate (*Punica granatum* L.) possess two groups of polyphenols that have health beneficial properties: anthocyanins (ATs, which also affect peel color); and hydrolysable tannins (HTs). Their biosynthesis intersects at 3-dehydroshikimate (3-DHS) in the shikimate pathway by the activity of shikimate dehydrogenase (SDH), which converts 3-DHS to shikimate (providing the precursor for AT biosynthesis) or to gallic acid (the precursor for HTs biosynthesis) using NADPH or NADP^+^ as a cofactor. The aim of this study is to gain more knowledge about the factors that regulate the levels of HTs and ATs, and the role of SDH.

**Results:**

The results have shown that the levels of ATs and HTs are negatively correlated in the outer fruit peels of 33 pomegranate accessions, in the outer peels of two fruits exposed to sunlight, and in those covered by paper bags. When calli obtained from the outer fruit peel were subjected to light/dark treatment and osmotic stresses (imposed by different sucrose concentrations), it was shown that light with high sucrose promotes the synthesis of ATs, while dark at the same sucrose concentration promotes the synthesis of HTs. To verify the role of SDH, six *PgSDHs* (*PgSDH1*, *PgSDH3–1,2*, *PgSDH3a-1,2* and *PgSDH4*) were identified in pomegranate. The expression of *PgSDH1*, which presumably contributes to shikimate biosynthesis, was relatively constant at different sucrose concentrations. However, the transcript levels of *PgSDH3s* and *PgSDH4* increased with the accumulation of gallic acid and HTs under osmotic stress, which apparently accumulates to protect the cells from the stress.

**Conclusions:**

The results strongly suggest that the biosynthesis of HTs and ATs competes for the same substrate, 3-DHS, and that SDH activity is regulated not only by the NADPH/NADP^+^ ratio, but also by the expression of the *PgSDHs.* Since the outer peel affects the customer’s decision regarding fruit consumption, such knowledge could be utilized for the development of new genetic markers for breeding pomegranates having higher levels of both ATs and HTs.

## Background

Fruits of pomegranate (*Punica granatum* L.) are well known for their health beneficial properties [1]. The health-promoting activities are attributed to the high concentrations of polyphenols found mainly in the fruit’s peels, which possess about 300-fold more polyphenols and 40-fold higher antioxidant activities than arils, the edible section [2–5]. These polyphenols act as scavengers of reactive oxygen species (ROS), and are associated with a reduction in stress-related chronic diseases and age-related disorders [2–6]. Previous studies showed that hydrolysable tannins (HTs) and anthocyanins (ATs) are the predominant polyphenols present in outer fruit peels [3–5, 7]. ATs are water-soluble pigments that are generally responsible for the red/violet/blue color of many fruits and flowers, including pomegranate arils and peels [3, 8]. These compounds are also produced in vegetative tissues and protect plants from environmental stresses, such as osmotic stress, drought, UV irradiation and low temperatures [9]. HTs are polyphenols in which some or all of the hydroxyl groups of the core carbohydrate moiety are esterified with phenolic acids, such as gallic acid (GA) or ellagic acid. HTs reportedly possess antioxidant, antimicrobial and anticancer activities in humans [10]. In pomegranates, HTs contain several unique compounds, including the α and β isomers of punicalagins and punicalins [11, 12]. Punicalagin isomers are most abundant in pomegranate peels and are responsible for more than 50% of the juice’s potent antioxidant activity [3, 13]. They also have health benefits such as induced growth inhibition and apoptosis in human prostate and papillary thyroid carcinoma cancer cells (e.g., [14, 15]).

**Fig. 1:**
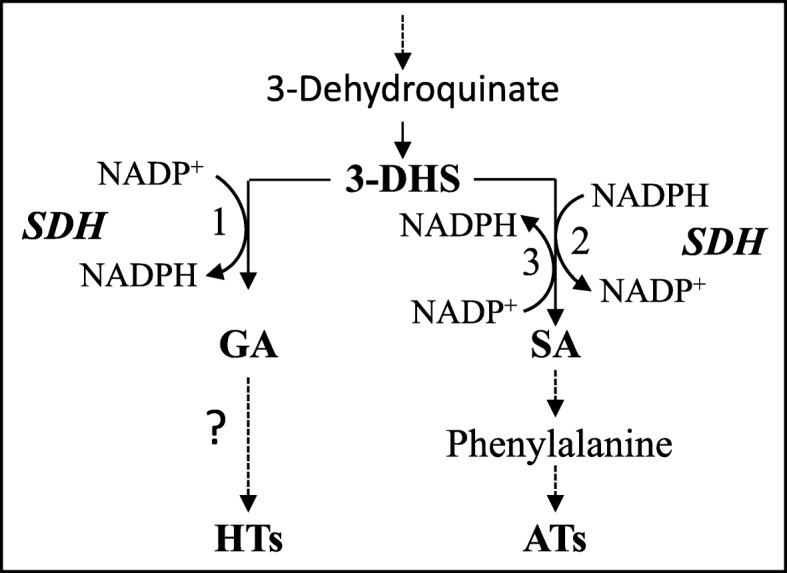
The role of shikimate dehydrogenase (SDH) in partitioning 3-dehydroshikimic acid (3-DHS) to gallic acid (GA) and hydrolysable tannins (HTs) vs. shikimic acid (SA) and anthocyanins (ATs) in plants. All three reactions are formed by shikimate dehydrogenase (SDH)

The biosynthesis of both ATs and HTs is related to the shikimate pathway (Fig. 1) [8, 16, 17]. ATs were formed from the phenylpropanoid pathway that begins with phenylalanine, an aromatic amino acid produced from the shikimate pathway [18]. HTs biosynthesis starts from gallic acid (GA) [16, 17, 19], which is suggested to be produced from an enzyme in the shikimate pathway, shikimate dehydrogenase (SDH) (EC 1.1.1.25) [16]. In plants, SDH is joined with dehydroquinate dehydratase (DQD) to form a bifunctional protein known as DQD-SDH, which catalyzes both the third (DQD) and fourth (SDH) reactions in the shikimate pathway [20, 21]. By analyzing the catalytic activity of SDH, this enzyme was shown to produce shikimate from 3-dehydroshikimate (3-DHS) in the presence of NADPH as a co-factor, whereas it formed GA in the presence of NADP^+^ (Fig. 1) [16]. A previous study showed that *Vitis vinifera* has four isozymes for DQD/SDH (EC 4.2.1.10/1.1.1.25), which have different affinity to the cofactors NADPH and NADP^+^ [22]. Moreover, analyses of enzyme activities suggest that *Vv*SDH1 has the ability to form shikimate, while *Vv*SDH3 and *Vv*SDH4 can produce GA [22].

Therefore, the redox state of the cell (NADPH/NADP^+^ ratio) is suggested to control the activity of SDH and the competitive partitioning of 3-DHS to shikimate and its associated metabolites (e.g., ATs), or GA and its derivatives (e.g., HTs). Consistent with this hypothesis, it was reported that a high level of ATs was correlated to a low amount of punicalagins (and *vice versa*) in the aril juice of most of the 29 pomegranate accessions analyzed [5]. However, such a correlation was not observed for ATs and HTs in fruit peels [5], apparently because both the inner and outer peels of these pomegranate accessions were analyzed together. Considering that ATs are present in the outer peels but not in the inner peels, it remains unclear whether an inverse correlation of ATs and HTs concentrations can be found in the outer peels of pomegranates where both compounds are abundant.

The objectives of the current study were to: (i) understand the distribution of the ATs and HTs in the outer peels of pomegranate; (ii) investigate the role of DQD/SDH in the accumulation of HTs and ATs in this fruit section; and (iii) reveal how environmental conditions could affect the expression of *DQD/SDH* genes and metabolite levels in the outer peels of pomegranate. Addressing these goals is important since high levels of ATs regulate the color of peels that attract consumers to purchase the fruit, while high levels of HTs are related to a reduction in some of the defects of the peels such as husk scald [23, 24]. Such data could also assist the breeder in producing fruits having high levels of both HTs and ATs.

## Results

### A negative correlation was detected between ATs and HTs in the outer peels of 33 pomegranate accessions

**Fig. 2:**
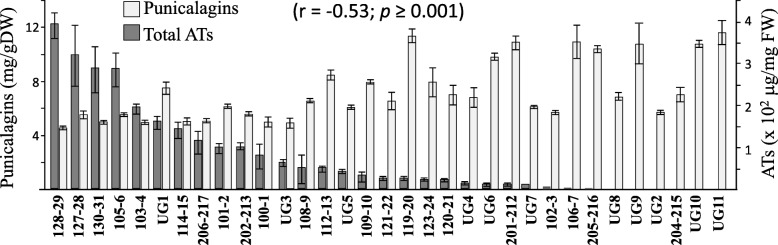
The levels of total anthocyanins (ATs) and punicalagins content in the outer peels of 33 pomegranate accessions. The data presented represent the mean ± SD of three biological replicates from each accession. The correlation analysis (r) was done by the Pearson test

To determine whether a negative correlation exists between the levels of HTs and ATs in the outer peels of pomegranate fruits, total ATs and the level of punicalagins were examined in the outer peels of 33 different pomegranate accessions (Fig. 2). Twenty-two of these accessions were collected from a farm in the Neve Ya’ar Research Center, ARO, which were also previously examined for the levels of these compounds in the arils [5]. In addition, 11 accessions collected from a growth farm in the Upper Galilee (UG accessions) were also analyzed. A peeler removed the outer peels of the fruit, then the contents of the two isomers of punicalagins were measured using HPLC, while the levels of total ATs were analyzed using a spectrophotometer. The levels of total ATs in the outer peels range from 0 to 380 μg/mg fresh weight (FW), while the levels of punicalagins range from 4.3 to 11.9 mg/g DW of dry peel (Fig. 2). A correlation analysis between total ATs and total punicalagins shows a significantly negative correlation (r = − 0.53; *p* ≥ 0.001).

### An inverse relationship between HTs and ATs in the outer fruit peel was observed when the ATs biosynthesis pathway was physically suppressed

It was observed that ATs accumulation in fruits is induced by sunlight to protect them from photoinhibition and photobleaching [25]. Indeed, pomegranate fruits developed in the tree shade have a less intense red color than those produced at the edge of the tree, which is exposed to sunlight. To examine the accumulation of ATs and HTs when AT biosynthesis is induced by sunlight, ATs and HTs profiles were determined in the fruits of PG100–1 and UG3, two accessions having a red-peel color, with and without exposure to sunlight. To prevent exposure to sunlight, seven fruits from PG100–1 and UG3 were covered with paper bags for about two months (August 1 to October 2). An additional seven fruits from each accession of similar size were marked as sun-exposed controls. All fruits selected were located in the outer canopy of pomegranate trees at similar positions on the tree.

**Fig. 3:**
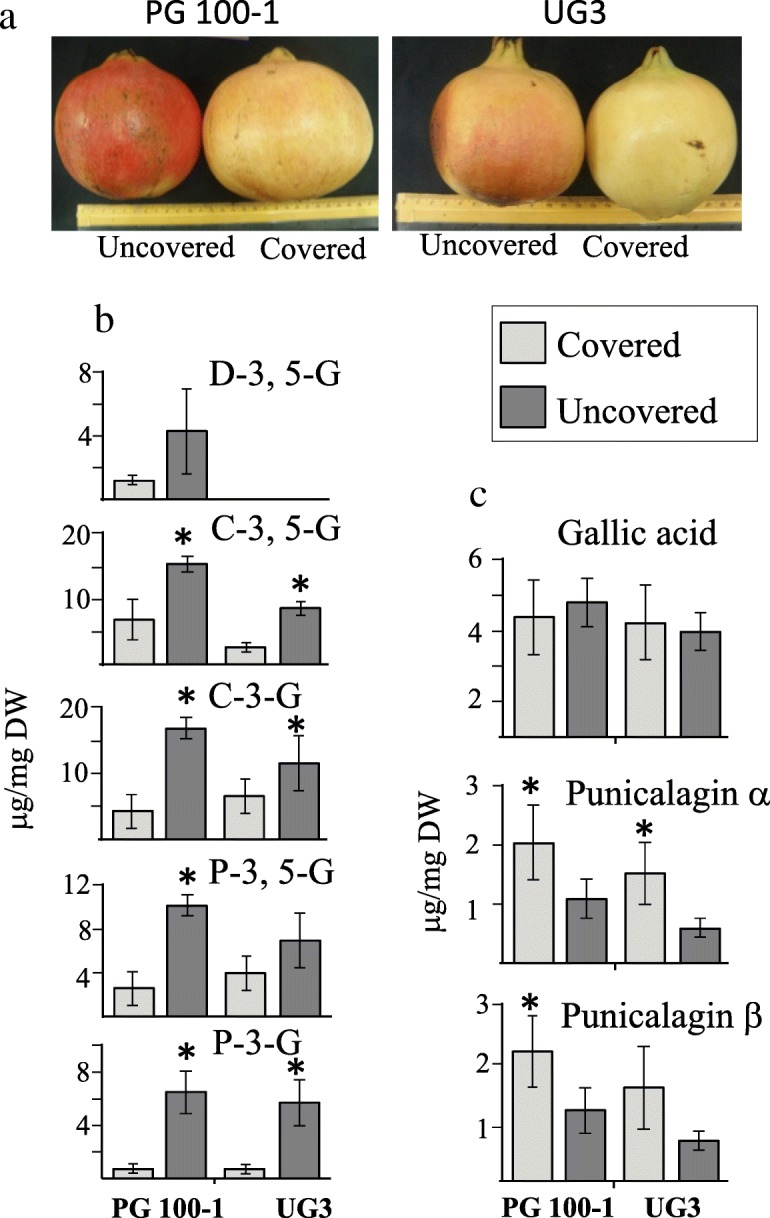
The morphological phenotype of representative fruits (**a**); the content of ATs (**b**); and the content of gallic acid and punicalagin isomers (**c**) in the outer peels of PG100–1 and UG3. The fruits were uncovered or covered with paper bags for two months during August and September. The peak areas of ATs, gallic acid and punicalagins were measured using HPLC, and their levels were calculated based on standards. The abbreviations for the ATs are: C-3-G and C-3,5-G, mono- and di-glucosides of cyanidin; P-3-G and P-3,5-G, mono- and di-glucosides of pelargonidin; and D-3,5-G di-glucoside of delphinidin. The data presented represent the mean ± SD of seven biological replicates from each accession. In each accession, comparisons were made between the two treatments using the student t-test and are indicated by asterisks (*p* < 0.05)

Peels of the covered fruits had a less red appearance than the sun-exposed fruits (Fig. 3a). The ATs profile was identified and quantified in the outer peels of PG100–1 and UG3 using HPLC-DAD-TOF-MS. Cyanidin 3-glucoside, cyanidin 3,5-diglucoside, pelargonidin 3-glucoside and pelargonidin 3,5-diglucoside were detected in both PG100–1 and UG3 accessions, while delphinidin 3,5-diglucoside was detected only in PG100–1 (Fig. 3b). In PG100–1, delphinidin 3,5-diglucoside was the only AT that did not decrease significantly in fruits placed in the bags compared to the control fruits (Fig. 3b). Among the four ATs detected, the levels of cyanidin 3-glucoside, cyanidin 3,5-diglucoside and pelargonidin 3-glucoside in the UG3 accession decreased significantly. In contrast to the reduced accumulation of ATs in the peels of the covered fruits, the levels of punicalagin isomers increased 2- to 2.4-fold in the covered fruits (which was statistically significant in PG100–1 and insignificant in UG3 for punicalagin β) (Fig. 3c). In these two accessions, the levels of GA did not alter substantially between sun-exposed and covered fruits (Fig. 3c).

### An inverse correlation between ATs and HTs was observed in peel calli cultures under different growth conditions

To further investigate the inverse relationship between HTs and ATs, and also to understand how abiotic stresses and different growth conditions affect the contents of ATs and HTs, we initiated an outer fruit peel culture from accession UG28, which has a red peel color [26]. The advantage of this culture is that it is an isolated system that does not receive molecular signals and metabolites from other tissues located outside the cells of the outer peel. To obtain sufficient tissues for the experiment, the peel cultures were grown for three months in MS medium containing 1% sucrose under dark conditions [26]. Subsequently, about one gram of calli was transferred to MS plates supplemented with different sucrose concentrations (1, 2.5, 5%, or 7.5%) that, at high concentrations, lead to osmotic stress, and the plates were placed in light or dark. After biweekly transfers for 75 days, calli grown in 1% sucrose in the dark appeared gray, while those that developed in the light had a green color resulting from chlorophyll accumulation (Additional file 3: Figure S1). Red spots and patches were observed in calli grown under light in 2.5% sucrose, which intensified in 5%, but decreased in 7.5% sucrose (Additional file 3: Figure S1). Calli grown in the dark showed a range of light gray to dark gray in media containing 1–7.5% sucrose (Additional file 3: Figure S1).

**Fig. 4:**
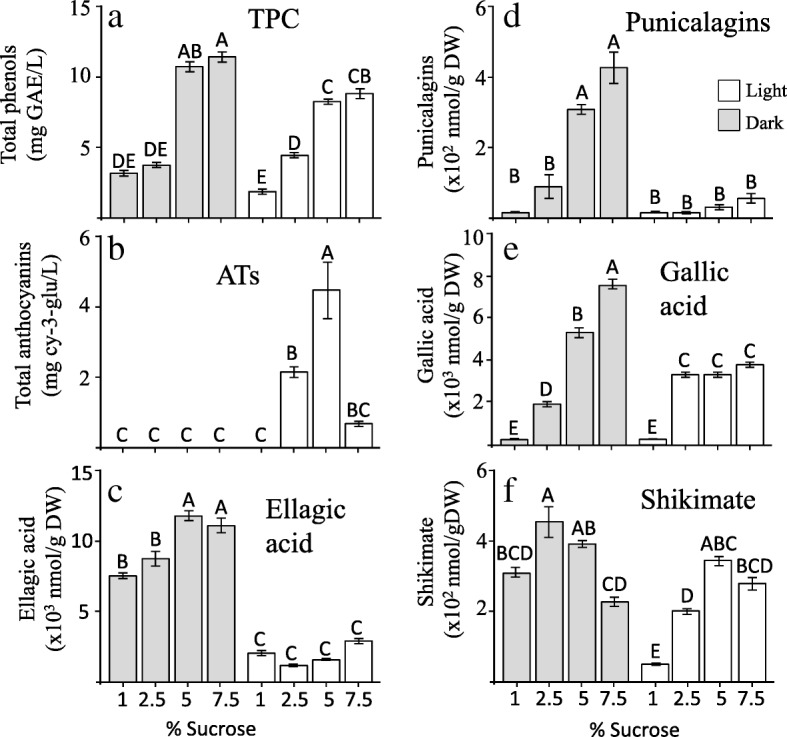
The levels of total phenols content (TPC) (**a**); total anthocyanins (ATs) (**b**); ellagic acid (**c**); punicalagins (**d**); gallic acid (**e**); and shikimate (**f**) in the calli formed from the outer peels of accession no. UG28. The calli grown in different MS media are supplemented with different concentrations of sucrose. The plates were placed in light and dark. The data presented represent the mean ± SD of four samples taken from four biological replicates. Statistically significant changes (*P* < 0.05, using one-way ANOVA) are identified by different letters

Calli that grew in 5% sucrose showed the highest dry weight among all treatments, and no significant differences were detected between calli grown in the light and the dark (Additional file 3: Figure S2-S3). Since sucrose at high concentrations causes osmotic stress, we also measured the water content within these samples, and showed that water content decreased from 2.5% sucrose with increases in sucrose in the media (Additional file 3: Figure S3). The levels of total phenol content (TPC) showed an increase at higher sucrose concentrations under both dark and light conditions, though significantly higher levels of TPC were detected in the dark than in the light in media containing 5 and 7.5% sucrose (Fig. 4a). The phenols profile was then analyzed using HPLC-DAD. This analysis identifies and quantifies the levels of ellagic acid, GA, shikimic acid and isomers of punicalagins that belong to HTs. The levels of these compounds and mainly punicalagins in calli were about 15-fold lower than the levels found in the original outer peels of accession UG28, as was also previously shown for calli originating from UG25 [26]. The results (Fig. 4) showed that dark increases the amount of ellagic acid relative to light, and it also induces the accumulation of GA and punicalagins at high sucrose levels. In the light, the levels of shikimate and ATs increased significantly from 1 to 5% sucrose concentrations in the media. However, in the dark, the levels of shikimate decreased significantly with an increase in sucrose concentration from 2.5 to 7.5%, while the levels of GA, ellagic acid and punicalagins increased significantly, strongly implying an inverse relationship between ATs and HTs at high sucrose concentrations in the media.

Five different types of ATs were identified in calli grown in the light using HPLC-DAD- TOF-MS, as we had detected in the fruit peels (Additional file 3: Figure S4). Except for pelargonidin 3,5-diglucoside, the four other ATs have the highest levels in 5% sucrose, which fits with the total ATs level. Cyanidin 3-glucoside and cyanidin 3,5-glucoside are the two main ATs in cultures. These results suggest that light/dark and sucrose concentrations significantly affect SDH activity, and thus determine the pathways that lead to HTs and ATs.

### Changes in levels of amino acids and the metabolic profiling of tissue culture cells

To understand the broader impact of varied growth conditions on metabolic profiles of peel calli, levels of aromatic amino acids (phenylalanine, tyrosine and tryptophan) derived from the shikimate pathway and other amino acids were determined using GC-MS. Phenylalanine and tryptophan levels were the highest in 1% sucrose in the dark and the light, and gradually decreased with an increase in sucrose concentrations. In contrast, the level of tyrosine tended to increase up to the highest level of 5% sucrose, mainly in the light (Additional file 3: Figure S5; Additional file 2: Table S1). The reduction in levels of phenylalanine and tryptophan is opposite to the elevations in TPC levels, suggesting that these two amino acids are used for the synthesis of various polyphenols compounds under osmotic stress.

Similar to these two latter amino acids, the levels of threonine, methionine and isoleucine decreased at 5 and 7.5% sucrose concentrations compared to 1–2.5% sucrose in the medium in the dark and the light, while valine and leucine decreased similarly but only in the dark. The levels of proline, lysine, serine and alanine increased with sucrose concentration in the medium. The levels of the latter two amino acids tend to increase in the light compared to the dark. The total soluble amino acids increased significantly at 2.5 and 5% sucrose concentrations in the medium, whose levels in the light were higher than in the dark (Additional file 2: Table S1).

**Fig. 5:**
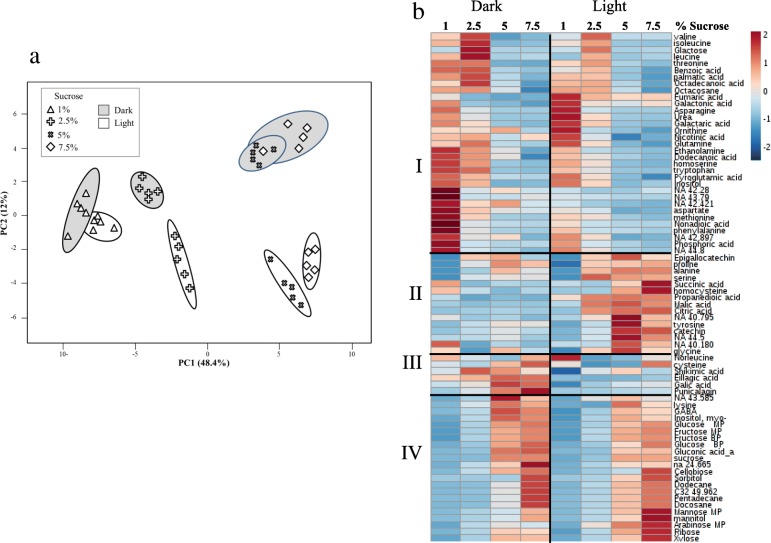
Graphical representation of changes in primary metabolite profiles of calli grown at different sucrose concentrations in the presence and absence of light. (**a**) Principal component analyses (PCA) applied to the primary metabolome set of 76 annotated and non-annotated (NA) metabolites analyzed using GC-MS. The data points are displayed as projections onto the two primary axes (eigenvectors). Variances explained by the first two components (PC1 and PC2) appear in parentheses; (**b**) Heat map of the 76 primary metabolites. The intensity of blue or red represents the value of the coefficient, as indicated on the color scale. Each column represents an average of four biological repeats. Four clusters were identified by different letters on the left

The effect of varied growth conditions on the levels of other metabolites was also investigated (Additional file 2: Table S2). A total of 76 metabolites were detected by GC-MS, including 10 metabolites as yet not annotated. A principal component analysis (PCA) was performed to reveal what factors mainly affected the accumulation of these metabolites. The interactions between dark/light and sucrose in the media tend to exhibit distinguishable differences. The PC1 representing 48.4% of the variance was due to the level of sucrose in the medium, while the PC2 representing 12% of the variance was mainly due to the light/dark conditions (Fig. 5a). This result implies relatively strong effects of these abiotic stresses on the primary metabolome of the cultures. A heat map analysis further revealed details about these metabolic changes. In total, four clusters of metabolites were detected (Fig. 5b). Clusters I (upper panel) and IV (lower panel) demonstrate the effect of high sucrose on the levels of the metabolites. The contents of metabolites in Cluster I decreased in response to high sucrose, while those of Cluster IV increased. The metabolites in Cluster I included several fatty acids, amino acids and sugars, while Cluster IV had higher levels of other sugars such as fructose, glucose, sucrose, arabinose and sugar alcohols, including sorbitol, mannitol and inositol, whose levels are known to increase under osmotic stress [27]. Clusters II and III (Fig. 5b) show the effect of light/dark on the levels of metabolites. Metabolites belonging to Cluster II increased in the light when sucrose content in the media increased, but those of Cluster III tended to increase with sucrose content in the media mainly in the dark. Cluster II (Fig. 5b) is characterized by metabolites that are intermediates of the tricarboxylic acid cycle (TCA), such as malate, succinate and citrate, but also by amino acids that are related to stress such as proline, serine, alanine and glycine (Fig. 5b). Cluster III contains HTs-related metabolites such as shikimate, GA, ellagic acid and punicalagins.

A correlation analysis with all the metabolites in the entire dataset showed that metabolites that tend to increase under osmotic stress (sugars and several amino acids, such as proline, GABA, lysine, serine, alanine, mannitol and inositol) were positively correlated (Additional file 3: Figure S6). However, they were negatively related to other metabolites such as aromatic and aspartate family amino acids, as well as to several amino acids whose levels were higher under low osmotic stress (Additional file 3: Figure S6).

### Identification and characterization of *PgSDHs* in pomegranate

**Fig. 6:**
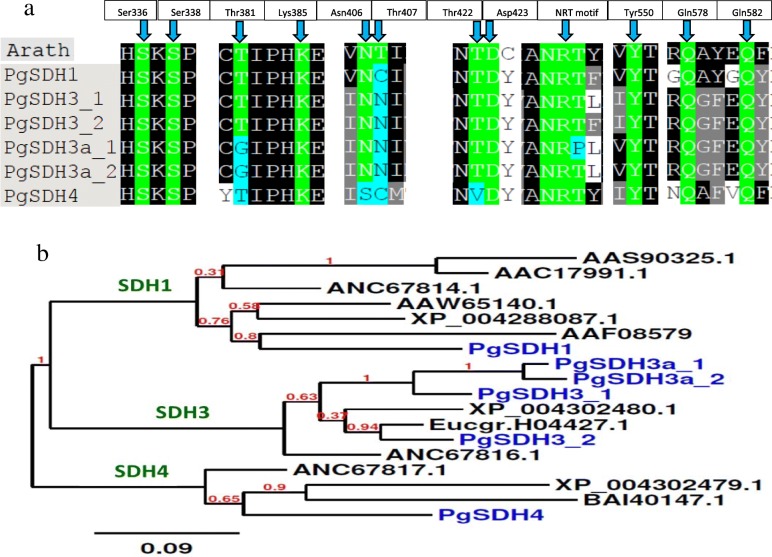
(**a**) Comparison of key amino acids in the SDH domain of DQD/SDHs from Arabidopsis (Arath) and pomegranate. Protein alignment was performed by the T-Coffee test. Numbering of amino acids is based on the DQD/SDH from *Arabidopsis thaliana* (AtSDH; [31]). Green color represents the active site residues of Arabidopsis protein. Identical amino acids at the respective positions in the pomegranate sequences are also highlighted in green; when they differ, they are highlighted in light-blue. (**b**) Phylogenetic tree constructed from *PgSDHs* and 12 plant SDH sequences available in public databases. *PgSDHs* are highlighted in blue. The clusters shown in the green letters are according to the classification proposed by Bontpart et al. [22]. A phylogenetic analysis was performed using the neighbor-joining method using phylogeny.fr, 0.1 substitutions per site. The sequences used for this analysis were of *V. vinifera* (Vv [22]; accession numbers ANC67814, ANC76816, ANC67817); *Juglans regia* (Jr, [16]; accession number AAW65140); *Nicotiana tabacum* (Nt, [32]; accession number AAS90325); *Diospyros kaki* (Dk, [33]; accession number BAI40147); *Fragaria vesca* (Fv, [34]; accession numbers XP_004302480, XP_004302479, XP_004288087); *Eucalyptus* sp. (Eg, [35]; accession number Eucgr.H04427.1); *Solanum lycopersicum* (Sl) accession number AAC17991; and *Arabidopsis thaliana* accession number AAF08579. Accession numbers correspond either to the NCBI or Phytozome 12 database

To investigate the role of DQD/SDH in determining the biosynthesis of GA/HTs and ATs, six homologs of *V. vinifera DQD/SDHs* were identified from the pomegranate data [8, 28–30] (see their transcript sequences in Additional file 1). Five types of SDH were previously characterized in *V. vinifera* [22] based on key amino acids in the SDH domain from *Arabidopsis thaliana* (AtSDH [31];). Thus, alignments analysis with defined amino from the *V. vinifera,* revealed that among the six PgSDH, PgSDH1 and PgSDH4 have only one isozyme, PgSDH3 have two isozymes (− 1 and − 2), and the other two PgSDHs have the PgSDH3 motif, together with one of the three motifs that characterize PgSDH5 [22] (Fig. 6a; Additional file 1). Because VvSDH2 exhibited very low ‘classical’ SDH activity compared to the other *Vv*SDHs [22], and since the sequence of PgSDH2 (closest to VvSDH2) was significantly truncated, *PgSDH2* was not examined further in this study.

The phylogenetic tree for these six sequences shows that the proteins encoded from these genes are grouped as expected into three clusters (Fig. 6b), as previously reported for *V. vinifera’s* SDHs [22]. Since the two PgSDHs having the PgSDH3 and PgSDH5 motifs are grouped in the same cluster of PgSDH3*,* we called these two sequences PgSDH3a (− 1 and − 2) (Fig. 6b). Blast and alignment analysis to their protein sequences indeed shows that they have high similarities (Additional file 1). Example is the comparison between PgSDH3–1 to PgHDS3a-1 that has 89.4% identity, and similarity of 92.7%.

The protein sequences of PgSDH1*,* PgSDH3*,* PgSDH3a and PgSDH4 have 65–75% similarity to AtSDH1, the only DQD/SDH in *A. thaliana*. The three PgSDHs are similar to their corresponding VvSDHs [22]. The VvSDH4 is 82.2% identical and has 90.7% similarity to PgSDH4; the VvSDH3 is 83.1% identical and has 91.9% similarity to PgSDH3–2, while VvSDH1 is 76.4% identical and has 87.8% similarity to PgSDH3 (Additional file 1). All of the PgSDHs, except for PgSDH3–2, do not contain transit peptide sequences at their N-terminus, suggesting that they function in the cytosol, whereas *PgSDH3–2* is expected to be located in plastids (Additional file 1).

**Fig. 7:**
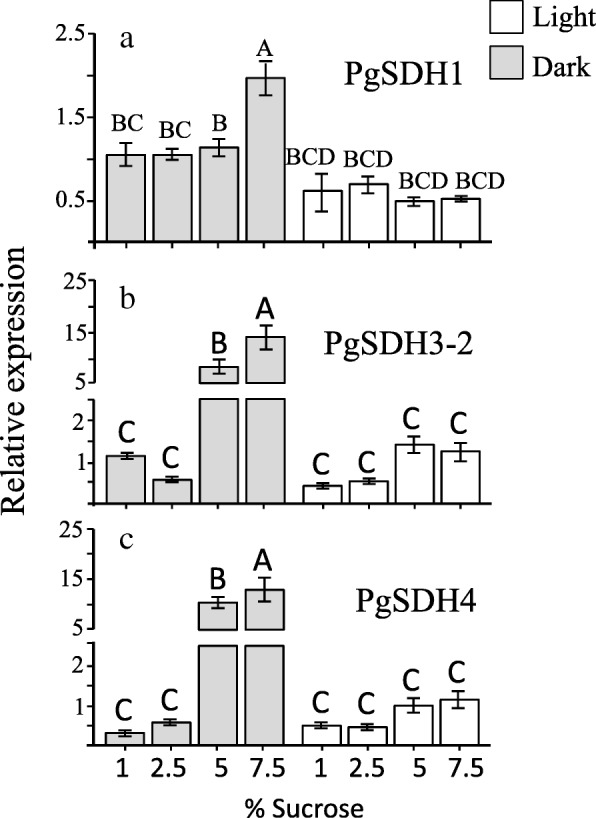
Reverse transcription-quantitative polymerase chain reaction (RT-qPCR) analysis of *PgSDH1* (**a**), *PgSDH3* (**b**) and *PgSDH4* (**c**) in calli grown at different sucrose concentrations in the presence and absence of light. The expression data were normalized to the housekeeping gene *PgRPSII*. The data are presented as the mean ± SD obtained from at least three independent measurements. The one-way ANOVA test was used to determine statistically significant differences (*P* < 0.05), which are identified by different letters

To determine the relationship between the expression of *PgSDHs* and the levels of HTs and ATs in fruit peel calli, transcripts of *PgSDH1*, *PgSDH3–2* and *PgSDH4* were analyzed using RT-qPCR (Fig. 7). RT-qPCR was also performed for *PgSDH3–1* and *PgSDH3a-1* but only in calli grown at 2.5 and 5% sucrose concentrations in light/dark conditions (Additional file 3: Figure S7). The expression level of *PgSDH3a-2* was not determined since its sequence is very similar to *PgSDH3a-1* (95.7% identity; 97.3% similarity) (Additional file 1), so we were unable to distinguish between these two isozymes, also because we not have the sequence of untranslated region of these genes. The results (Fig. 7; Additional file 3: Figure S7), show that in general, the expression levels of *PgSDH3, PgSDH3*a and *PgSDH4* tended to increase with an increase in sucrose in the medium. These elevations were significantly higher in the dark at 5 and 7.5% sucrose concentrations than in the light (Fig. 7). *PgSDH1*, however, exhibited the highest expression when grown in 7.5% sucrose in the dark, indicating that its expression is higher in the dark than in the light. However, its expression level did not significantly alter in media containing 1 to 5% sucrose (Fig. 7). The expression levels of calli placed in 7.5% sucrose in the dark were about 2-, 15- and 35-fold higher compared to the levels detected in calli placed in 1% sucrose in *PgSDH1*, *PgSDH3a-2* and *PgSDH4*, respectively (Fig. 7). This suggests that the osmotic stress triggers the expression levels of these genes, and this effect is emphasized more in the dark than in the light. When the expression level of these *PgSDHs* was compared to the levels of HTs and ATs, it was shown that *PgSDH3(− 1,-2*), *PgSDH3a (− 1,-2)*, and *PgSDH4* have a relatively similar pattern to the levels of HTs and TPC, unlike *PgSDH1*.

## Discussion

This study suggests that an inverse relationship exists between the levels of HTs and total ATs in the outer peels of most of the accessions of pomegranate fruits. This supported by three lines of evidence: (i) the inverse correlation between HTs and ATs in the outer peels of 33 pomegranate accessions (Fig. 2); (ii) the inverse correlation between punicalagins and ATs in fruits with and without sunlight exposure (Fig. 3); and (iii) increased ATs and decreased GA, ellagic acid and punicalagins in fruit peel calli grown in the light compared to the dark under osmotic stress (Fig. 4). In addition to the negative correlation between HTs and ATs, an opposite trend was found between the GA and shikimate in fruit peel calli grown in the dark on MS medium supplemented with 2.5–7.5% sucrose. These observations suggest a critical role of DQD/SDH in partitioning the common precursor 3-DHS between the synthesis of GA (precursor for HTs biosynthesis) and shikimate (providing precursors for ATs biosynthesis). It also suggests that the biosynthesis pathways leading to ATs and HTs competed for their common precursor, 3-DHS. However, there are many other shikimate pathway-derived metabolites besides ATs, such as flavonoids, condensed tannins and lignins (reviewed by [18, 21]), which may at least partially explain why the correlation between HTs to ATs in the outer peels of the 33 accessions (although it is significant) is only *p* > − 0.53.

Although our results are in accordance with previous studies indicating that GA derived from the shikimate is the main precursor for HTs synthesis [16, 18, 22], we cannot exclude the possibility that other pathways also contribute to GA synthesis. It was suggested that GA could be produced from syringate, intermediate metabolites of polyphenols such as lignin and ATs [36, 37]. Indeed, it was proposed that GA is produced from syringate in pomegranate leaves at early stages of development in the inner seed coat and pericarp, while in the other organs and in late stages of these latter organs, GA is mostly produced from shikimate [30]. The finding that there are at least two pathways that leads to the synthesis of GA in pomegranates hints to its important roles. GA is produced in plants that do not produce HTs, also implying its additional role in plants in general. Indeed, GA is reported to be the most potent antioxidant among simple phenols [36], and it has been identified as an allelopathic compound secreted from roots inhibiting the root growth of neighboring competing plants [38]. Furthermore, GA can convert to compounds such as gallic acid-glucoside (used as a signal during development), to epitheaflagallin and to epitheaflagallin 3-O-gallate [35]. Its levels are also dependent on its degradation, since GA is used to produce pyruvate and oxaloacetate, both of which are critical components involved in multiple pathways, including the TCA  cycle [38]. The finding that the levels of GA remain relatively stable in covered and non-covered pomegranate fruits (Fig. 3) while the levels of punicalagins changed suggests that GA plays additional roles also in the pomegranate’ outer peels, and therefore an inverse relationship between its contents and punicalagin is not trivial.

### The putative source of NADPH/NADP^+^ for *PgSDH* activity

It was previously suggested that the ratio between NADPH/NADP^+^, the cofactors of SDH, regulates its activities to GA (when NADP^+^ is abundant) or shikimate (when the level of NADPH is high) [16]. NADPH can be formed in plant cells from several different pathways in the chloroplast, cytosol and mitochondrion 39, 40]. The major pathways that maintain the cytosolic NADPH are the oxidative pentose phosphate pathway (OPPP), in which NADPH is produced in the conversion from glucose-6-phosphate to 6-phosphogluconolactone, and the conversion from 6-phosphogluconate to ribulose-5-phosphate. In mitochondria, NADPH can be formed by the activity of the cytosolic NADP-isocitrate dehydrogenase using carbon derived from mitochondrial citrate, from a triose-phosphate shuttle suggested to export reductant from the chloroplast in the light, and from the citrate valve that exports NADPH from mitochondria under photo-respiratory conditions ([39] and references therein). In chloroplasts, NADPH is derived from the reaction of ferredoxin-NADP^+^ reductase in the last step of the electron transport chain of photosynthesis [34, 38].

Among the above-mentioned pathways for the generation of NADPH, OPPP is found to play a major role during stress responses in plants [41]. OPPP can operate in the plastids and the cytosol. In plastids, both the OPPP (in the dark) and the Calvin cycle (in the light) produce erythrose-4-phosphate, which, together with glycolysis-derived phosphoenolpyruvate, act as precursors for the shikimate pathway [18]. Moreover, it was strongly suggested that the increased NADP^+^/NADPH ratio that occurs mainly in stress enhances OPPP activity, providing precursors required for the increased demand of phenolic metabolites that are essential during stress to protect the plants [42, 43]. Our results are in accordance with those studies. The observation that the TPC and HTs levels increased in calli that are grown in the dark under osmotic stress further implies that the NADP^+^/NADPH ratio increased, which eventually led to enhanced synthesis of polyphenols and specifically HTs from GA to protect the calli from the osmotic stress.

### The role of *PgSDH* in the branch point leading to GA and shikimate

In accordance with the pomegranate dataset, six *PgSDHs* were identified, two of which that are called *PgSDH3a* have new combinations of amino acids in the sequences that are defined at the active site of this enzyme (based on [22, 31]), and, as far as we know, they have not yet been reported in other plants’ SDHs. Youan et al. (2018) also reported on the existence of six *PgSDHs* isozymes in the pomegranate genome [29]. Although they did not publish the sequences, they showed that three are in tandem and are duplicated and located in a 100-kb region, and two showed decreased expression during fruit development, consistent with the observation that the levels of HTs also decreased during pomegranate fruit development [8]. Two other SDH genes are also found in tandem [29]. These findings are in accordance with the high identity between *PgSDH3* and *PgSDH3a*, which are most probably those that are in tandem. Further studies could clarify this issue.

The study of *Vv*SDHs showed that *Vv*SDH1 exhibited the highest SDH activity forming shikimate, but did not convert 3-DHS to GA [22]. *Vv*SDH1 was highly expressed both before and after the beginning of the ripening stage, showing its relative stability in contrast to *Vv*SDH3 and *Vv*SDH4, whose expression levels were significantly higher in immature berry tissues [22]. Similar to *Vv*SDH1, *PgSDH1* expression is relatively stable (except at 7.5% sucrose in the dark when its level increased significantly), suggesting that *PgSDH1* could be active to form shikimate and possibly use NADPH formed by the OPPP as it is predicted to be located in the cytosol.

It was proposed that the shikimate pathway in plants is regulated primarily at the transcriptional level [20]. It was also reported that the expression of shikimate pathway genes responded to abiotic and biotic stresses [43]. Indeed, the transcripts of *PgSDH3–2* and *PgSDH4* increased 15- and 35-fold, respectively, in calli grown in 7.5% sucrose medium compared to calli placed in 1% sucrose (Fig. 7). This elevation occurs in the dark, where significantly higher concentrations of GA and HTs accumulated, suggesting that the activity of *PgSDH3*, *PgSDH3a* and *PgSDH4* may lead to the synthesis of phenols required for protecting the cells from osmotic stress. Since *PgSDH3–2* has a plastid transit peptide, it may mainly use the NADP^+^ that accumulates in the dark in plastids, while *PgSDH3–1*, *PgSDH3a* and *PgSDH4* used this co-factor that mainly accumulates during stress [40].

The results obtained in the current study are in accordance with other studies showing that members from SDH groups 3 or 4 are highly expressed in plants having high amounts of HTs such as strawberry and eucalyptus, or galloylated flavan-3-ols like in tea plants, grape and persimmon [22, 44]. By contrast, species without SDH3 and SDH4, such as Arabidopsis, tobacco, tomato and orange, do not produce HTs and galloylated tannins [22]. Measurements of their activities in vitro in grape berry indeed showed that *Vv*SDH3 and *Vv*SDH4 exhibited high activity in forming GA and lower ‘classical’ SDH activity forming shikimate [22]. In addition, the formation of GA and gallotannins such as galloylated flavan-3-ols in grape berry during fruit development is synchronized with the relative expression patterns of *Vv*SDH3 and *Vv*SDH4. Furthermore, over-expression of *Vv*SDH3 in grapevine hairy roots resulted in an increase in GA and galloylated tannins [22]. The role of SDH3 in the synthesis of HTs was also recently verified in a study carried out on *Quercus ilex* [42]. The observation that the protein sequences of *PgSDH* are about 82% identical to *V. vinifera* SDHs, and that no differences were observed in the amino acid residues in the active site of the enzymes, suggests that the *PgSDHs* have similar activity as *Vv*SDHs.

Taken together, the findings of the current study are in line with those described in grapes, whereby *PgSDH1* is a more constantly expressed enzyme that is active with NADPH to form shikimate, while *PgSDH3*, *PgSDH3a* and *PgSDH4* can be induced by stresses and are operated more with NADP^+^ to form GA and thus HTs. Moreover, the current results also point to the observation that the expression levels of *PgSDH3*, *PgSDH3a* and *PgSDH4*, which significantly increased under osmotic stress in the dark, are major factors regulating the accumulation of GA and HTs, and not only the NADPH/NADP^+^ ratio.

### The effect of light, dark and sucrose concentration on the formation of HTs and ATs

Previous studies showed that abiotic stresses can affect the production and accumulation of ATs and HTs. The levels of ATs increase, for example, in stresses such as high light, salt, osmotic and drought [45–47]. The levels of HTs increased significantly in the peels of pomegranate fruits of 11 accessions grown under hot and dry environmental conditions compared to those grown in a Mediterranean climate [48]. In addition, osmotic stress formed by high sucrose levels in the medium was suggested to influence polyphenol metabolism and tannins production in tissue cultures of several plants [49–51]. A relatively high sucrose content also led to a higher content of tannins in the calli of *B. verbascifolia, Tamarix tetrandra* and *Quercus acutissima* grown in the dark, and only residual HTs were detected in the light [49–52], as is reported also in the current study. These findings further support our outcomes that GA is produced mainly in the dark.

In this study, light was found to be a critical stimulus regulating ATs in outer peels (Fig. 3) and in the calli (together with high sucrose content) (Fig. 4). On the other hand, light irradiation and sucrose treatment appears to inhibit HTs production in the calli, while at the same sucrose concentration in the dark, the levels of HTs significantly accumulated. Together, these studies suggest that a combination of osmotic stress under light or dark conditions can significantly affect the levels of ATs and HTs*.*

These abiotic conditions also affect the levels of other metabolites in the calli. The reduction of phenylalanine and tryptophan at higher sucrose levels in pomegranate calli suggest that they are used as a substrate for polyphenols that accumulate under these conditions (Fig. 4a). Indeed, the levels of GA, ellagic acid and punicalagins increased significantly when the levels of sucrose increased in the medium (Fig. 4). Comparable results were also reported in Arabidopsis culture exposed to salt. The levels of shikimate increased together with shikimate-related metabolites such as in coniferin [53, 54]. In addition, cell suspension of grape berry exposed to strong light and high temperature (40 °C) showed high elevations of metabolites downstream to the phenylpropanoid pathway as resveratrol and its derivatives piceid, epigallocatechin and coumaroylated forms of peonidin and cyanidin, dihydroflavonol glucosides and quercetin-3-*O*-glucoside [55].

Besides the more abundant HTs, the pomegranate calli show that the levels of proline, serine and alanine, as well as total amino acids, together with several sugars and sugar alcohols (sorbitol, mannitol and inositol) also increased under osmotic stress conditions. Accumulations of these metabolites are well known to occur under osmotic stress to protect plants [55, 56]. Also, the contents of intermediates of the TCA cycle also increased significantly. This finding is similar to those reported for Arabidopsis culture exposed to salt [53, 54]. Cell suspension of grape berry exposed to high temperature and light stress also showed high elevations of sugars, serine, alanine, proline, glutamate and metabolites of the TCA cycle [53]. However, the levels of threonine, methionine and isoleucine decreased under osmotic stress in the pomegranate calli. These amino acids are known to degrade to metabolites that feed the TCA cycle under stress to gain more energy [57].

## Conclusions

Taken together, the results of this study strongly suggest that the biosynthesis of HTs and ATs competes for the same substrate, 3-DHS, which is used by DQD/SDH to form GA or shikimate (Fig. 1). The relative activity of DQD/SDH for shimikate and GA is regulated not only by the ratio of NADPH and NADP^+^, but also by the expression of *PgSDH3, PgSDH3a* and *PgSDH4*. Light with high sucrose promotes the synthesis of ATs in calli, while dark at the same sucrose concentration promotes the synthesis of HTs. Both groups of metabolites are used to protect the calli from these abiotic stresses/conditions.

Our results provide deeper insight on the factors that regulate the levels of HTs and ATs in the outer peels of pomegranate fruits. The importance of the outer peels is not just attributed to the fact that the customer’s decision to consume the fruit is based on the first impression of the phenotype of the peels, mainly color and ATs content, but also to the nutritional value of pomegranate juice (that has high levels of HTs, which are usually extracted into the juice in the industries). In addition, high levels of HTs in the peels are related to less physiological defects such as husk scald [24]. The knowledge obtained in this study could be utilized for the development of new genetic markers for breeding pomegranates having high contents of ATs and HTs in the same fruits.

## Materials and methods

### Plant materials

Thirty-three pomegranate accessions were chosen for this study. Twenty-two of these accessions were collected from a farm in the Neve Ya’ar Research Center, ARO [registered in the Israel Gene Bank for Agriculture Crops (IBG, http://igb.agri.gov.il)] [58], and 11 accessions were collected from the Havat Hamataim operated by Northern R&D, Israel. The accessions differed in their peel colors. The fruits of the different accessions were harvested between September and November when the fruits were fully matured according to commercial practice. The fruits were transported via a ventilated car to the laboratory, where their outer peels were removed by a peeler. The peels were frozen in liquid nitrogen and kept at − 80 °C for further analyses. For the peel-tissue culture, the immature pomegranate fruits (3–4 cm in diameter) from accession UG28 were collected in June from Havat Hamataim.

### Determination of HTs and ATs

HTs and ATs were analyzed using a high-performance liquid chromatography-diode array detector (HPLC-DAD). HPLC was performed with an UltiMate 3000 system consisting of a solvent delivery module LG-980-02, a pump LPG-3400SD, an autosampler WP53000TSL, a column compartment TCC3000SD and a detector DAD3000 UV/vis. The column was a Hydro-RP-C_18_ Synergi column, 3 mm × 100 mm, and particle size = 2.5 μm (Phenomenex, UK). Ten μl of water extracts of calli or outer peels were injected into the column. Elution was performed at a flow rate of 0.8 mL/min using a gradient of water/formic acid (99:1 v/v) (A) and acetonitrile (B) (Sigma-Aldrich, Rehovot, Israel.): 100% A and 0% B at time 0, 96% A and 4% B at 3.6 min, 85% A and 15% B for 22 min, 50% A and 50% B at 23 min, 20% A and 80% B from 25 to 28 min, 100% A and 0% B at 32 min until 35 min. The metabolites were detected at 360 nm for punicalagins, 280 nm for other HTs, 320 nm for GA, and 520 nm for ATs. Identification and quantification of punicalagin isomers, GA and ellagic acid were achieved by comparing the retention time and standard curves of the authentic standards (Sigma-Aldrich, Rehovot, Israel).

For ATs determination, a peak assignment was performed by the software based on UV/vis absorbance spectra and the retention times of ATs standards. The standard library was constructed from cyanidin 3,5-diglucoside and cyanidin 3-glucoside (Sigma Aldrich, St. Louis, MO). Each of these standards (50–100 μg/mL methanol) was injected separately, and the data acquired by the photodiode array detector with the 3D feature were incorporated into the system’s ATs standard library. Relative standard deviation for the retention times in repetitive runs was in the range of 0.4–1.9%. Individual ATs were quantified from the corresponding chromatogram peak area calculated by the software. Calibration curves (linear, R2 = 0.99) were constructed with standards for each of the ATs at four concentrations (0.01, 0.10, 0.25 and 0.50 μg/mL).

### Determination of TPC, total ATs and metabolic profiling

Total phenolics and total ATs were determined as previously described [5]. Profiling of amino acids and other primary metabolites was conducted as previously described [59].

### Outer peel tissue culture

Immature pomegranate fruits of accession UG28 were washed with soap and water, the colored fruit peel was removed using a peeler, and small peel segments (measuring 1.0 ± 0.2 cm^2^) were cut and surface-sterilized as previously described [26]. The outer peel segments were placed in half-strength Murashige and Skoog (MS) medium containing 1% sucrose and 1 mg/L of 6-benzylaminopurine (BAP). The cultures were maintained in a growth room at 22 °C in the dark. The explants were transferred every two weeks to fresh medium for 11 weeks. After the initial development of the calli, the explants were removed; the calli continued to grow on the same medium and continued to be transferred every two weeks for an additional 10 weeks. The calli were then transferred to MS with 1, 2.5, 5% or 7.5% sucrose, and half of the plants at each sucrose concentration were transferred to light under a 16 h light/8 h dark photoperiod with light intensity of 50 μmol m^− 2^ s^− 1^ [26].

The cultures were weighed every two weeks after being transferred to a fresh new medium until the end of the experiment in order to monitor growth rates (Additional file 3: Fig. S2). At the end of the experiment, the fresh and dry weights were measured.

### Peels and calli extracts

Peels and the calli induced from the pomegranate outer peels were harvested, frozen in liquid nitrogen, and kept at − 80 °C until being dried with a lyophilizer, after which they were ground with a mortar and pestle into a fine powder. For water extraction, 50 mg of the dried tissue was mixed with distilled water at a ratio of 1:19 (v:v). The mixture was shaken for 40 min at 50 °C and then centrifuged for 20 min at 10,000 x g to remove the tissue debris. The supernatant was filtered through a 0.22 μm polytetrafluoroethylene (PTFE) filter and transferred to new tubes.

### mRNA extraction and reverse transcription-quantitative polymerase chain reaction (RT-qPCR) analysis

RT-qPCR was used to determine the expression levels of the three *PgSDH* genes in the calli grown in the dark and the light. Fifty mg of lyophilized dry calli were extracted using the Spectrum plant total RNA kit (Sigma). cDNA synthesis and qPCR procedures were carried out according to a previous study [60]. To normalize variance among samples, we used the constitutive-ribosomal Protein S (*PgRPSII*) gene as an endogenous control [7]. The values presented are means of three biological replicates, each with three technical replicates. The primers used for analyzing the expression of *PgSDHs* and *PgRPSII* are shown in Additional file 2: Table S3.

### Phylogenetic analysis

A phylogenetic tree analysis was made of selected dehydroquinate dehydratase/shikimate dehydrogenases taken from dicots. This phylogenetic tree was constructed from the six *PgSDHs* sequenced in this study and the 12 sequences available on public databases (NCBI) using Clustal Omega Phylogenetic options.

### Statistical analysis

Statistical analysis of the metabolite and gene expression data was performed using JMP version 8.0 (SAS Institute Inc., Cary, NC). Significant differences between treatments were calculated according to the Kramer HSD-Tukey test (*p* < 0.05). The Pearson test was used for the correlation analysis. Phylogenetic trees were created using the MAFFT version 7 [56] with the WAG model and a 1000 bootstrap. The SDH protein sequences were aligned using Clustal Omega.

## Supplementary information

**Additional file 1. ***Punica granatum* SDH sequences. (A) The transcribed RNA sequences of *PgSDHs*; (B) Alignment analysis between the six PgSDHs; (C) Alignment analysis between the six *PgSDHs* and the *Vv*SDHs; (D) Blast analysis between *PgSDH4* and *Vv*SDH4, and between *Vv*SDH3 and *PgSDH3–2*; E. Alignment analysis between the four isozymes of *PgSDH3* and *PgSDH3a*.

**Additional file 2: Table S1.** Soluble amino acid contents in pomegranate peel calli. **Table S2.** GC-MS metabolite dataset of pomegranate peel calli. Values are relative peak areas normalized to the norleucine internal standard. **Table S3.** Oligonucleotides used for the amplification and measurement of the expression of genes by qRT-PCR analysis.

**Additional file 3: Figure S1.** Phenotype of calli formed from the outer peels of accession UG28 grown on MS plates supplemented with different concentrations of sucrose under light (upper panel) and dark (lower panel) conditions. **Figure S2.** Fresh weight (gr) of pomegranate peel calli grown for 75 days (collected at five time points) on MS plates supplemented with different sucrose concentrations (1, 2.5, 5, 7.5%) under light and dark conditions. The media were replaced every 15 days. The values presented are the average ± SD of four biological replicates. **Figure S3.** The fresh weight, dry weight and water content in calli formed from the outer peels of accession UG28 grown on MS plates supplemented with different concentrations of sucrose. The plates were placed in the light (upper panel) and the dark (lower panel), and measurements were taken after 75 days. The data presented represent the mean ± SD of four samples taken from four biological replicates. Statistically significant changes (*P* < 0.05, using two-way ANOVA) are identified by different letters. **Figure S4.** Anthocyanin (AT) accumulation in calli formed from the outer peels of accession UG28 grown in the light on MS plates supplemented with different concentrations of sucrose. The peak area of each metabolite was measured using HPLC-DAD and the five ATs were detected. The levels of the mono- and di-glucosides of cyanidin (C-3-G and C-3,5-G), mono- and di-glucosides of pelargonidin (P-3-G and P-3,5-G), and mono-glucoside of delphinidin were measured based on standards of C-3-G and C-3,5-G s. The data presented represent the mean ± SD of four biological replicates. Statistically significant changes (*P* < 0.05, using two-way ANOVA) are identified by different letters. **Figure S5.** The content of three aromatic amino acids in calli formed from the outer peels of accession UG28 grown on MS plates supplemented with different concentrations of sucrose. The plates were placed under light or dark conditions. The data presented represent the mean ± SD of four samples taken from four biological replicates. Statistically significant changes (P < 0.05, using two-way ANOVA) are identified by different letters. **Figure S6.** Correlation matrix and cluster analysis of 76 primary metabolites detected in calli grown on MS plates supplemented with different concentrations of sucrose and under light or dark conditions. Each square of the heat map indicates Pearson’s correlation coefficient for a pair of compounds. The intensity of blue or red represents the value of the coefficient, as indicated on the color scale. Hierarchical clusters are represented in the cluster tree. **Figure S7.** Reverse transcription-quantitative polymerase chain reaction (RT-qPCR) analysis of *PgSDH3–1* and *PgSDH3a-1* in calli grown at 2.5 and 5% sucrose concentrations in the light or dark. The expression data were normalized to the housekeeping gene *PgRPSII*. The data are presented as the mean ± SD obtained from at least three independent measurements. The one-way ANOVA test was used to determine statistically significant differences (*P* < 0.05), which are identified by different letters.

## Data Availability

The datasets supporting the results of this article are included within the article and its additional files.

## Abbreviations

3-DHS: 3-dehydroshikimate
ATs: Anthocyanins
BAP: 6-benzylaminopurine
DQD: dehydroquinate dehydratase
DW: dry weight
FW: fresh weight
GA: gallic acid
HPLC-DAD: high performance liquid chromatography-diode array detector
HTs: hydrolysable tannins
MS: Murashige and Skoog
OPPP: oxidative pentose phosphate pathway
PCA: principal component analysis
PTFE: polytetrafluoroethylene
ROS: reactive oxygen species
RT-qPCR: reverse transcription-quantitative polymerase chain reaction
SDH: shikimate dehydrogenase
TCA: tricarboxylic acid cycle
TPC: total phenols content
