# Supplementary material for: Elucidating the role of shikimate dehydrogenase in controlling the production of anthocyanins and hydrolysable tannins in the outer peels of pomegranate
Source: BMC Plant Biol. 2019 Nov 6;19:476. doi: 10.1186/s12870-019-2042-1 (PMC6836501; doi:10.1186/s12870-019-2042-1)
Supplement: Supplementary file 1 — Additional file 1. Punica granatum SDH sequences. (A) The transcribed RNA sequences of PgSDHs; (B) Alignment analysis between the six PgSDHs; (C) Alignment analysis between the six PgSDHs and the VvSDHs; (D) Blast analysis between PgSDH4 and VvSDH4, and between VvSDH3 and PgSDH3–2; E. Alignment analysis between the four isozymes of PgSDH3 and PgSDH3a. [file 12870_2019_2042_MOESM1_ESM.docx]

**Additional file 1**

**Additional Files-** *Punica granatum* SDH sequences. (A) The transcribed RNA sequences of PgSDHs; (B) Alignment analysis between the six PgSDHs and the Arabidopsis SDH; (C) Alignment analysis between the six PgSDHs and the VvSDHs; (D) Alignment analysis between SDH proteins belonging to PgSDH3; (E) Blast analysis between PgSDH4 and VvSDH4; VvSDH3, and PgSDH3-2; and PgSDH1 and VvSDH1;

**Additional File A.** The transcribed RNA sequences of the PgSDHs

| **Gene name** | **Sequence ID** | **Sequence** |
| --- | --- | --- |
| PgSDH1 | GBGR01003923.1 | CAATAGCTTTCCGCCTAAGGTGACATCCTCTTTCACTTGCTTAAATATGGAGGCTGCAGCAATGAGGCAGAATTCAACGTTAATCTGTGTTCCTATAATGGGGGACTCCATTGATAAGATGGCAATTGATATGGATAGAGCCAAAGCCAGTGGAGCTGATCTTGTGGAGATTAGGCTGGACTGTTTAAAGACATTCAGTCCAAATGAGGATCTCAAGACTATAATGAAGGCTTCTCCTCTGCCGACACTAATTACATACAGACCAAAATGGGAAGGGGGACAGTATGAAGGTGATGAAAACAAGAGACTGGATGTTCTTCGGTTAGCTGTGGAGTTGGGTGCTGACTATGTTGATGTCGAGCTTCAGGTTGCTCGTGAGTTTAATGATTCCATAAGTGGCAAGAAACCTCAGAATTGCAAGGTCATAGTTTCTTCTCACAATTATGAGTCCACTCCATCTATTGAGGATCTTGGCAATCTTGTTGCAAGAATACAAGCTGCTGGCGCTGATATAGTGAAAATTGCAACTACTGCCTTAGATATTACTGATGTTGCACGAATCTTTCAAACAATTGTGCACTCCCAATATCAGGTTCCAATTATAGGACTAGTGATGGGAGAAAGGGGTTTGATTTCACGAGTTCTTTGTGCCAAATATGGTGGGTATCTCACTTTTGGTACTCTTGACTCTGGAGTAGTCTCAGCTCCTGGCCAACCTACTGTCAAGGATTTGTTGGATTTATACAACTTCAGGAGCATAAGGCATGACACTAAAGTGTTTGGCATTATTGGGAAGCCAGTCGGGCACAGCAAGTCGCCTTTTTTATACAATGAAGCCTTCAAATCAGCTGGTTTTAATGGGGTCTACTTGCACTTGTTGGTGGATGGCGTAGCAAACTTTCTCCAGACTTACTCATCCACTGATTTTACTGGATTCAGCTGTACCATTCCCCACAAGGAGGAAGCAGTTAAATGCTGTGATGAGGTTGATCCAGTTGCTAAGTCGATTGGAGCTGTTAATTGCATAATCAGGAGAGCAAGCGATGGGAAGTTACTTGGGTACAACACCGACTATGTTGGTGCAATTTCTGCTATTGAGGATGGATTGCAAGGTTCTCACGGTGCTAGCAACTCCACTTCTTCACCTTTATATAACAAGCTATTTATTGTGATCGGTGCTGGTGGTGCTGGGAAGGCTCTTGCTTATGGTGCGAAGGAAAAAGGAGCAAGAGTGGTTATTGCAAATCGAACTTTTGAGAGAGCAAAAGAACTTGCGGACACCATTGGAGGAGATGCACTATCTCTTGCTGATTTAGAGAATTTCCACCCTGAGGATGGCATGATTCTTGCAAATACAACATCTATCGGAATGCAACCGAAAGTTGATGAAACACCTGTCCCCAAGCAAGCTCTGAAGCACTATGCACTAGTCTTTGATGCTGTTTACACCCCAAAAATCACCCGACTTCTGAGGGAAGCTGAAGAATGTGGGGCCACAACTGTATCTGGGATCGAGATGTTCATTGGACAGGCTTATGGACAATACGAGAGGTATACTGGGTTGCCTGCACCAAAGGAACTCTTCAGGAAGCTTATGTCAACAAAATGAGGGATCCGCTTGCTTCATGTAATTGTGATATCTTTACATCTGTATGTTTGAACTTATCCTTTTCCCCTCGAGGGTCATGTAACTTGTTATTCGAGGAAATCACAACACTCATCTATATTGTTTTGAACTCAATACTTTCAAAGCAAGCAAGGAAAGATAAAAATCCTGATGGCTTGATCTTGAGTTTCCTTGCTACTCACTGCCAAATGACGTTCTGAATCTGTAACTTTTGCCATGGAGAAGTGAGCTCCTTTCCTTGCAGTTTTGTCGAAATAGCCCAACTATTGGCCAGCCTCCATTTTAGAATTCGTTTATGGGATTTTGGATGCATTTCTTCAAGCGAAAGATTGTATTAGAGGGAGGGAGGATGACTGTGTAGCTCCTTCTCCTCCAAATCCAAACATAGCAGCTCCTCTTCCATCATATTGACTGCATCAGAGAGAGGGAGGATGACTGTGAAACTCCTCCAAATCCAAATACAGCAGCTCCTCTTCCATCAAATTGCCCTCCGTCTGTCACCAATAATCGCCACAGGAACACGAACCCCCTTTAAAGTGGTGGAAGCGGTTATTGTCTCTCAAAATTATC |
| PgSDH3a_1 | OWM62975.1 | \| ATGAGCACGAGCAGCCGCACTTTACTGTGCACTCCCCTGATGGGGACCAC \| \| --- \| \| CGTGGATCAGATGCTGACTGAGATGAGGAAGGCCAAGGAGATCGGGGCAG \| \| ATGTCGTCGAGGTTCGGTTGGACTGCTTGAGGAAGTTCAATCCTCACCAG \| \| GACCTTGAGATACTCATCAAACGGTCTCCTCTGCCCACCCTTATCACTTA \| \| CAGACCACTCCGGGAAGGTGGTCAGTATGACGGAGACGAGAACAAGAGAC \| \| AAGATGCTCTACGTTTGGCAATGCACCTTGGCGCCAGCTACGTTGATATC \| \| GAGCTTGAGGTTGCTTATGAGTTCATTAATTCCATTCGCGTAAAGAAGCC \| \| AGAGAACTTCAAAGTCATAGTCTCCTCCCACAATTTCCACGAAACTCCTT \| \| CCTCTGAAGCCATTGGGAAGCTTGTAGCTATGATACAAGCTTCTGGGGCT \| \| GATATCGTCAAGGTAGCAACGACCGCTTTGGACATTACGGACTGTGCACG \| \| GGTTTTCCAAATGATGGTCCAATCTCAGATTCCAACAATAGGAATTTCTA \| \| TGGGAGAGAGGGGTCTTATTTCCCGGATACTCAGCCCAAAGTTTGGCGCA \| \| TATCTCACTTACGGTGCCCTCGAGGCCAGTGCTATATCAGCTCCGGGTCA \| \| ACCTCTGGCAAAGGATTTGTTGGATCTCTATAACTTCCGACTCATAAGAC \| \| CAGACACCAAAGTGTACGGCGTCATCAGCAAGCCCGTGGGCCACAGCAAG \| \| AGCCCTCTTCTGTTTAATGCTGCATTCAAATCGGCTGGTCTCAATGCAGT \| \| CTATGTGCACCTTTTGGTGGATGATGTCAAGAAGTTTTTCGAGACATATT \| \| CGGCTGCAGACTTTGTTGCCGGATGCAGTTGCGGGATCCCTCACAAGGAA \| \| GTTGCACTTAAATGCATGGACGAGATTGACCCCATTGCCAAGAAAATTGG \| \| TGCTATTAATAACATCGTGAGGAGACCTGATGGGACGCTGGCTGCTTTCA \| \| ACACAGACTATATCGGTGCTATCTCTGCCATCGAGGATGGACTGCGAGAG \| \| CTAAATGGTGCGACTCCCGCAGTGGGCTCGCCCTTGGCAGGTAAATTGTT \| \| TGTCGTCCTCGGAGCTGGAGGAGCTGGCAAGGCACTTGCCCATGGAGCTG \| \| CCCAAAAGGGTGCTCGGATTGTTGTTGCCAATCGTCCACTGGAACAAGCC \| \| AAGGAACTTGCTGATAAGGTCGGCGGACAAGCTATGACTCTTGATGAAGT \| \| CGCCAGTTTCCACCCTGAAGACGAGATGGTTCTTGCCAATGCTACCTCTG \| \| TTGGCATGACGCCAAAGATCGATCAGACCCCTATACCCAAACAAGCTTTG \| \| AAAAATTACTGCCTAGTTTTTGATGCCGTCTACACACCAAAAGACACCAG \| \| GCTCCTCAGAGAAGCCAGAGAGTCGGGAGCCTTCATCGTGTATGGAACCG \| \| AGATGCTGATCCGACAGGGCTTTGAGCAGTACAAAAATTTCACGGGATTG \| \| CCAGCGCCGGAAGAGTTGTTCAGGATGGTCATGATGAAGCACGCATAA \| |
| PgSDH3a_2 | OWM62976.1 | \| ATGAGCACGAGCAGCCCGACTTTACTGTGCACTCCCCTGATGGGGACCAC \| \| --- \| \| CGTGGATCAGATGCTGGCTGAGATGAGGAAGGCCAAGGAGATAGGGGCGG \| \| ACGTCTTGGAGATACGGTTGGACTGCTTGAGGAAGTTCAATCCTCACCAG \| \| GACCTCGAGATCCTCATCAAACGGTCTCCTCTGCCCACCCTTATCACTTA \| \| CAGACCACTCCGGGAAGGTGGTCAGTATGACGGAGACGAGAACAGGAGAC \| \| AAGATGCTCTACGTTTGGCAATGCACCTTGGCGCCAGCTACGTTGATATC \| \| GAGCTTGAGGTTGCTTATGAGTTCATTAATTCCATTCGCGTAAAGAAGCC \| \| AGAGAACTTCAAAGTCATAGTCTCCTCCCACAATTTCCACGGAACTCCTT \| \| CCTCTGAAGCCATTGGGAAGCTTGTAGTTATGATACAAGCTTCTGGGGCT \| \| GATATCGTCAAAGTAGCAACGACCGCTTTGGACATTACGGACTGTGCACG \| \| GGTTTTCCAAATGACGGTCCAATCTCAGATTCCAACAATAGGAATTGCTA \| \| TGGGAGAGAGGGGTCTTATTTCCCGGATACTTAGCCCAAAGTTTGGCGCA \| \| TATCTCACTTACGGTGCCCTCGAAGCCAGTGCTATATCAGCTCCGGGTCA \| \| ACCTCTGGCAAAGGATTTGTGGGATCTCTATAACTTCCGACTCATAAGGC \| \| CAGACACCAAAGTGTATGGCGTCATCAGCAAGCCCGTTGGCCACAGCAAG \| \| AGCCCTCTTCTGTTTAATGCTGCTTTCAAATCGGCCGGTCTCAATGCAGT \| \| CTATCTGCACCTTTTGGTGGATGATGTCAAGAAGTTTTTCGAGACATATT \| \| CGGCTGTAGACTTTGTTGCCGGATGCAGTTGTGGGATCCCTCACAAGGAA \| \| GTTGCACTTAAATGCATGGACGAGATTGATCCCATTGCCAAGAAAATCGG \| \| TGCTATTAATAACATCATGAGGAGACCTGATGGGACGCTGACTGCTTTCA \| \| ACACAGACTATATCGGTGCTATCTCTGCCATCGAGGATGGACTGCGAGAG \| \| CTAAATGGTGCAACTCCCGCAGTGGGCTCGCCCTTGGCAGGTAAATTGTT \| \| TGTCGTCCTCGGAGCTGGAGGAGCAGGCAAGGCACTTGCCTATGGAGCTG \| \| CCCAAAAGGGTGCTCGGATTGTTGTTGCCAATCGTACACTTGAACGAGCC \| \| AAAGAACTTGCTGGTAATGTCGGCGGGCAAGCTATGACTCTTGACGAAGT \| \| CGCCAGTTTCCACCCTGAAGACGAGATGGTTCTTGCCAATGCTACCTCTG \| \| TTGGCATGACACCAAAGATTGATCAGACCCCTATACCCAAGCAAGCTTTG \| \| AAAAATTACTGCCTAGTTTTTGATGCCGTCTACACACCAAAAGACACCAG \| \| GCTCCTCAGAGAAACCAGAGAGTCCGGAGCCTTCATCGTGTATGGAACCG \| \| AGATGCTGATCCGACAAGGCTTTGAGCAGTACAAAAATTTCATGGGATTG \| \| CCAGCGCCGGAAGAGTTGTTCAGGATGGTCATGGAGAAGCACGCATAA \| |
| PgSDH3_1 | OWM62977.1 | \| ATGGGAAGCCTTTCGCTTGCGGTATCTGATATTCAAACGAGCACGAGCGG \| \| --- \| \| GATGAGGGGTAGCCCGACTTTACTGTGCACTCCCCTGATGGGGACCACCG \| \| TGGATCAGATGCTGATTGAGATGAGGAAGGCCAAGGAGATCGGGGCAGAT \| \| GTCGTCGAGGTTCGGTTGGACTGCTTGAGGAAGTTCAATCCTTTCCAGGA \| \| CCTCGAGATCCTCATCAAACGGTCTCCTCTTCCCACCCTTGTCACTTACA \| \| GACCATTCTGGGAAGGCGGTCAATATGATGGGGACGACAACAAGAGACAA \| \| GATGCTCTACGTTTGGCTATGCACCTGGGCGCCAGCTATGTTGATATCGA \| \| GCTTGAGGTCGCTTATGAGTTCATTAATTCTATTCATGGAAAGAAGCCAG \| \| ACAACTTCAAAGTCATTGTCTCCTCCCACAACTTCCACAATACTCCATCT \| \| TCTGAAGCCATCGGGAATCTTGTAGCTAGAATACAAGCTTCTGGGGCTGA \| \| CATCGTTAAAGTAGCAACAACCGCTTTGGACATTACGGACTGTGCACGTG \| \| TTTTCCAAATAATGGTCCACTCTCAGATTCCAACAATAGGAATTGTTATG \| \| GGAGAGAGGGGCCTTATTTCCCGGCTACTTAGCCCAAAGTTCGGAGCATA \| \| TCTCACTTATGGTGCCCTCGATACTGGTGCTATATCAGCTCCGGGTCAAC \| \| CATTGGCAAAGGATTTGTTGGATCTCTATAACTTCCGACTCATAAGGCCA \| \| GACACTAAAGTGTATGGCATTATCGGGAAGCCTGTAGGCCATAGCAAGAG \| \| CCCTCTTCTGTTTAATGCAGCTTTCAAGTCGGTCGGTCTCAATGCAGTCT \| \| ATGTGCACCTTTTGGTGGATGACGTCGAGAAGTTCTTCGAGGCCTATTCG \| \| GCTGTAGACTTTGTTGCTGGATGCAGTTGCACAATCCCTCACAAGGAAGT \| \| TGCAGTTAAATGCATGGGCGAGATCGACCCCATTGCCAAGAAAATCGGTG \| \| CCATTAATAACATCGTGAGGAGACCTGATGGGACGCTGACTGCTTTCAAC \| \| ACGGACTATATTGGTGCTATCTCTGCGATCGAGGATGGACTACGAGAGCT \| \| AAATGGTGCAACTCCCGGAGTGGGCTCGCCTTTGGCGGGTAAATTATTTG \| \| TTGTACTTGGAGCTGGCGGAGCTGGGAAGTCACTTGCCTATGGAGCTGCC \| \| CAAAAGGGTGCTCGGGTTGTTGTTGCCAATCGAACACTGGAACGAGCCAA \| \| AGAACTGGCTGATAAGGTAGGAGGGCAAGCTATGACTCTTGATGAAGTCG \| \| CTAGTTTCCACCCGGAAGACAGGATGGTTCTTGCCAATACTACCTCTGTT \| \| GGCATGAAACCAAATGCCGATGGGACCCCAATACCCAAGCTAGCTTTAAG \| \| ACATTACTGCCTAGTTTTTGATGCCATTTACACGCCAAAAGACACCAGGC \| \| TCCTCCGAGAAGCCAGAGAGTCTGGAGCTATCATTGTGTACGGAACTGAG \| \| ATGCTGATCCGACAGGGCTTCGAGCAGTACAAAAATTTCACGGGTTTGCA \| \| AGCCCCGGAAGAGTTGTTCAGGACGCTCATGGAGAAGCACGCGTAG \| |
| PgSDH3_2 | OWM85406.1 | \| ATGGGGAGGGAGGTCTCAGAGAGCTGCGTGGAGAGCCTTCTCATCGAGAT \| \| --- \| \| GGTCTCTTCGTACTGCAACCGCTTCTACGCCGACAAGCCGGAGCTTGCCG \| \| CCCGGAGAATCGAGGCCATCGGCTATCAGGTCGGGCACCAGCTCTCCGAG \| \| AGATATACGATGGAACGGCCTCGGTTCATTGATCACTTGGAAGCAATCAA \| \| GTTCATATGCAAGGACTTCTGGTCAGAGATCTTCAAGAAGCAGATAGACA \| \| ATCTGAAGACTAACCACAGAGGAACTTTTGTGTTGCAAGATAATCGCTTC \| \| CGCTGGCTTTCACGCATGTCGATCGAGTCGTCACCTGAGACGACGGGTTC \| \| ATCGCAAGATGGTGAGGACAAGGCAGCACAAGCAATGATCCCACAATACC \| \| AGTCATACCCAACAGGTTACTCTACAACAATGGCCAGCGTCCCGTTCACC \| \| ATTTCCGACCTCCAAACGAGCGGCAGCGGGTTCCGGAGCAGCCCGACCCT \| \| GCTATGCACTCCGCTGATGGGGACGACGGTGGACCTGATGCTGATAGAGA \| \| TGGGGAAGGCTAAGGAGATCGGTGCGGACCTCCTGGAGATCCGGCTCGAC \| \| TGTCTGAGGGCCTTCAACCCTCGCCAGGATCTTGACATTCTCATCAAGCA \| \| GCCTGTTTGGGAAGGAGGCCAATATGAAGGCGACGAGAACAAGCGACAGG \| \| ACGCATTGCGTCTGGCAATGCAGCTCGGTGCTAACTACGTTGATGTCGAG \| \| CTTGAGGTTGCCCATGACTTCAACAATTCTATTCAGGGAAAGAAGCCCGA \| \| CAGCTTCAAAGTCATAGTCTCCTCTCACAACTTCCACAACACCCCCTCGG \| \| CCGAGGCCATCGGGAATCTTGTCGCGAGAATCCAAGCCACCGGTGCCGAT \| \| ATCGCGAAGATTGCAACCACGGCACTCGACATCACCGACTGCGCACGCAT \| \| TTTCCAGATAACTGTCCACTCTGAGATTCCAACTATCGGAATTGTTATGG \| \| GAGAGAGAGGCCTGATCTCTCGTATTCTCAGCCCCAAGTTCGGCGGATAT \| \| CTCACTTATGGTGCGCTTGAGGCCGGCGCAATATCTGCTCCAGGACAACC \| \| GACAGCAAAGGACTTGCTAGAACTGTACAACTTCAGACTCATTAGGCCCG \| \| ACACCAAGGTGTACGGCATCATCGGGAAGCCTGTGGGCCACAGCAAGAGC \| \| CCTCTTCTCTTCAATGCAGCATTCAAGTCGGTCGGTCTCAATGCAGTCTA \| \| TCTGCATTTTCTTGTTGACGATGTCGAGAAGTTCTTCAAGACCTACTCGG \| \| CTGTGGACTTTGCCTCAGGGTGCAGTTGCACGATCCCGCATAAGGAGGCT \| \| GCACTTAAATGCATGGATGAGATTGACCCGATTGCTAAGAAAATTGGTGC \| \| TATCAACAACATCGTAAGAAGACCAGACGGGACATTGACTGCATTTAACA \| \| CCGATTACATCGGTGCCATTACTGCTATCGAGGATGGCGTGAGAGAATTG \| \| AATGGTGCGACCCCATCCGCCGTCTCTCCCTTGGCTGGGAAACTGTTTGT \| \| TGTCCTCGGGGCCGGTGGGGCTGGAAAATCACTAGCTTATGGAGCAGCAC \| \| AGAAGGGGGCACGAGTTGTTGTCGCCAACCGTACATTCGAGAGAGCGAAG \| \| GAGTTGGCAGAGAAGGTTGGGGGAAAAGCTCTAACTTTGGAGGAAGTTCA \| \| GGACTTCCACCCGGAAGAAGGGATGATCCTTGCGAACACAACATCCGTTG \| \| GGATGAAACCAAAGATCGATGAGACCCCCCTAGCTCAGCATGCTTTGAAA \| \| CACTACTCCGTGGTGTTCGATGCCATTTACACCCCGAAGGATACCCGTCT \| \| GTTAAAGGAAGCTAGAGAGACCGGAGCTGTCATCGTTTATGGGACAGAGA \| \| TGTTGATACGCCAAGGCTTCGAGCAGTACAAGAACTTCACGGGTCTGCCT \| \| GCACCGGAAGAACTGTTCAGGCAACTTATGGAGAAACACGCGTAG \| |
| PgSDH4 | OWM85405.1 | \| ATGATATGTGCCCCGTTAATGGCGGATTCGGTCGATCAGATGTTGAGTCT \| \| --- \| \| AGTGAGGAGCGCCAAAGACCAGGGCGCTGACTTGGTCGAGCTTCGGTTGG \| \| ACTTCTTGAAGAGCTTCAGCCCCAGACAAGACCTCGAGACCCTTATCCAG \| \| CAGAGCTCCTTGCCCACTCTTGTCACTTTCAGACCAAAATGGGAAGGTGG \| \| ACAATATGAAGGTGATGAAAAAGCTCGACTTGACGCGTTGCGTCTAGCTG \| \| TGGAATTAGGATCTGCTTATGTTGATGTTGAGCTTAAGGTAGCCAATGAG \| \| TTTTTCAACTCCGTAAAAGGGAAGAAGCCTGAAAAGGTTAAAGTGATCGT \| \| TTCTTCTCACAATTATGAAAAGACTCCATCTTCCGAGGAACTCGGCAACC \| \| TCGTTGCAAGAATTCAGGCTACAGGAGCTGACATTGTGAAGATTGCTACA \| \| ACTGCCACGGACATCACTGACAATGCACGGATCTTTCAAGTACTCGCGCA \| \| TTCTCAAGTCCCAACAATAGCAATTGCTATGGCTGACAGAGGTTACATAT \| \| CAAGGATACTTTCTGGGAAATACGGAGCTTTCCTGACCTTTGGGACGCTT \| \| GAGGCAGGAGTGGAGTCTGCTCCTGGACAGCCCACTTTAAAGGATTTATT \| \| GGACCTCTATAACTTCCGACAAATTGGTTCTGATACCAAAGTCCATGGTG \| \| TTATTGGAAACCCGATTGGCCACAGCAAAAGTCCTCATTTATACAACTCA \| \| GCATTTAAATCAGTCAACTTCAATGGGATCTACCTACCTTTGTTGGTTGA \| \| TAATGTTGCAAATTTCATAAGCACATTCTCTTCTCCAGATTTTGTCGGAT \| \| ACAGTTACACTATTCCTCACAAGGAAGCAGGACTTAAATGCTGTGACGAG \| \| GTCGACCCAATTGCACAGGCTATAGGAGCTATTAGCTGCATGACCAGAAG \| \| GCCGGATGGATCGATGATGGGGTACAATGTCGACTACCTCGGGGCCATAG \| \| CAGCTATTGAGGAAGCACTCCGAGCCCGGGAAGGTACAAATGGAGCTGCT \| \| TCGCCCTTAGCTGGTAAACTCTTCGTTGTCATTGGCGCTGGTGGAGCTGG \| \| AAAGGCACTTGCTTATGGTGGTTACGAGAAGGGCGCAAGAGTCATGGTCG \| \| CAAACAGGACATATGACAAAGCAAAAGAACTTGCTAGCAAAGTCGGAGGG \| \| AAAGCCATTACCCTTGAAGAACTCGAGAATTTCCACCCAGAAGAGGGGAT \| \| GATCCTTGCCAACACCACATCTGTTGGGATGAAACCGAACATTGATAGGA \| \| CACCCTTAGCTAAGAAAGCTTTGAGCCACTATTCTCTGGTTTTTGATGCA \| \| ATCTACACCCCGAAATTGACCCGACTCCTGAGGGAAGCTCAAGAGTGTGG \| \| AGCCACCCCTGTGTACGGAACCGAGATGTTCATCAACCAGGCCTTTGTAC \| \| AGTTTGAGAGGTTTACAGGGATGCCAGAGCGGTCGATTGCCGCCTCGGCT \| \| GTCTCTTGTTCTCAGGTAGAATATCTCTGCTCCGATCGTGCTGTTTATGT \| \| CGATATCTGTTTATTCTCCTGCCGATTGCTTGTGGAATTGGTTAAAGTTC \| \| TCTCTGCTTGTGGATCTTTTGGTCGAGGAGTTGAATTCCTCCTGGGGATC \| \| GATTATGGAGAAGTGTCTCGTTCGGTGGATATAGCAGGCGGATTGGGGCC \| \| TGAAATGAAACTTGCTTGCTCGACTGATTGGGGGCAAAAGCAAGTTGCTT \| \| GCATACGTGAGGAGGAAGAAAGTATGGATGAAGCTGAAGCAGATGGGGAG \| \| GCCTTCACCGAACTACAGCCTGTGGAGCAAGAGGACAAGAAGGAAGCTGA \| \| AACACCTGTAGAGCCCTCCACAGTGTTACAGACCATAGAGCAAGCTGAAA \| \| CACCCAGTGCATCCAATAAGTATTTGGTTGCGAAGCAAGGGAGTAAAGAG \| \| GAAACTGAAACACCACCGGAGACCTTCCCTGAGTTGCAGATTGTGGAGCA \| \| AGAGACAAAGAATGAGTGTGAAATGCCATCAGAGGCATCCGTTCGGTCTC \| \| GACCTCCAGAAAAGAAAGCTGCAAATTCATTGAAAGCCAGGGCTAAGATG \| \| GTCAATAAATCTGCCGCCAAGAGCTTCTTGAATGCCAAAAATGGGAAAGC \| \| TGCTCTGAAAGTGCAGAAGAAAATAGTGAAGAAGAACAAAGGAGCACCCC \| \| TGGTTATCAATGAAGGTAGCAAGGATGGTGGAGAGACTGGGGGCGCCAAA \| \| GAAGAGTATGTGGAGCAAGAGAACAAGAAGGAAGCTGAAACTCCCACGGA \| \| GGTCTCCACCATGTTTCACAGTTCTGAGAAAGGGACTGGAAAGGAAGCTG \| \| AAATGCCTGCTACCTCTGTCCAGGCTGAAGCTATATCTCCCCAAAAGAGA \| \| ACTCCAAATTCACTGAAAGTTGGAGCCAAGATAGTAAAGAAAGTTTCGCC \| \| CAACAAATTATGGAATACTGCAGGCAGGAAAGCTGCTCTTAACAAAGTTC \| \| AGAAGAAAATAGTAAAGAACAAATCGCTGGTTCTCAATAGCAGTGGTAAG \| \| AATTCAGGAGAAACCGCCAATGACAATACTCTGACAGTTAGTGTGACTGA \| \| TGTTAAGGACAATCCAGGGAATAAAAGAAAGGATACTAGCCACAATGACG \| \| ATAAGAATGGCAGGGAGCCTGCTTTGGGTGACATAAATGATAGTGGAAAG \| \| CAAATCGTGCAGGGGAAAGGTGGGAAGACTAAAAGGAGACAGAGGAAACA \| \| CGAGAATAACCAAGGTCGCAGCTCAAATGATGGAGGCGAAAATCCGAAGA \| \| AGAATGAGAGGCTTGAGAGGGAGGAAATGGACCAAGGGAAAAAGGGGAAG \| \| GAGGAGAAAGAAAAAATTGGGGGTTTGATCTTATTGTGCAGCACAAAGAC \| \| CAAGCCAGATTGTTTGAATTATCGTGTCATGGGTGTTCCTGAGAGCCAAA \| \| AAGATATGGTCTTGGCGGTCAAACCTGGTCTGAAGTTATTCTTGTATGAT \| \| TTCGATCTCAAGCTTATGTATGGGATCTATAAGGCATCCTCTGCTGGAGG \| \| CAAGAAACTTGAGCCCCGAGCTTTTGGAGGGGCCTTTCCCTATCAGGTGC \| \| GGTTTAGAACTCATATTGATTGCTTCCCTCTTCCTGAGACCATTTTTAAG \| \| AAGGCGATGATGGAGAATTTCATCACGAAACACAAGTTCAAAACAGAACT \| \| TACCTTTCAGCAGGTCAGAAAGCTCACAGAACTTTTCCGACCAGTGGAGA \| \| TCCACCCAAGTGTCCAACCACGACCAGACAAGTTTCCTCCCCGCTCTGCA \| \| AGAAAGGAAAGAGCCAGAGATAGGGATGCCCGTTTGAGCATGAGTGAATC \| \| TCAGCATCATTCACAAAGGGAAACAGTGCGAACTGATCCTTACGCAGGGA \| \| GGGATGAGAGGAGGTATCCTTTGTTGGCTCGTGAAGGAGATCGGCTCACT \| \| GCAAATCGAGAGGTGAGAGCGGAACCTCCTCGAGAAATGTTCCTGACAGA \| \| GGCAGACTACCGAGCTTATGGTCTTCGAGGCTGGAGAAGGTACTCAGATC \| \| TCCCCAGTCGTGCAGCTCCTCCATTAGACCCGTACTGGGAAGATTACGAG \| \| AAGAGGAGACAACCCGATTTAGTTTACCGGGATGCTGTGCCTGCACGTAG \| \| AGAGTATGTCTATGCGAATCCCAGTTACTCGGACTATAGAGACTGTCAGA \| \| CTTACTCTTCTTATGATGCTAGAAGGGAATATCCTATCGTCCATCGGTAT \| \| CCAGATTATCAAAGAAGAGCTGCTGTCTCATCTTCTGAGGCTTACCCGAC \| \| TGAGTCCAACCGGGACTTGCGTAGGAGAGAGAGAGATCCTGTTGATAGAA \| \| TCTATACAGAATATGATATGAGACGTTACCACATCGCGGATCCCGATCTT \| \| GCGGCCCTGCCTGTTTCTTCCCGCTATTCTTTTGCGGGTCCAAGGTAA \| |

**Additional File B.** Alignment of SDHs from *P. granatum* and *A. thaliana.* Protein alignment has been performed using T-Coffee. Amino acids identical and similar to Arabidopsis template are highlighted in black and grey, respectively. The numbering of amino acids is based on the DQD/SDH from *Arabidopsis thaliana* (AtSDH; [31]). Since N-terminal signal peptide was not included in Arabidopsis structure determination by Singh and Christendat, 2006 it was omitted from the alignment. Amino acids highlighted in bright green represent the active site residues of Arabidopsis protein. Identical amino acids at the respective position in the pomegranate sequences are also highlighted in bright green, and in aqua blue when they are different the identical amino acids at the respective position in the pomegranate sequences, and when they are different from AtSDH. Orange and blue bars above the sequence highlight the two functional domains (DQD and SDH), respectively.

Arabidopsis_SDH 79 ------------------------------STEMEIGSHDIVKNPSLICAPVMADSIDKM
PgSDH1 1 -----------------------------------MEAAAMRQNSTLICVPIMGDSIDKM
PgSDH3_1 1 -----------------------MGSLSLAVSDIQTSTSGMRGSPTLLCTPLMGTTVDQM
PgSDH4 1 ----------------------------------------------MICAPLMADSVDQM
PgSDH3a_2 1 ---------------------------------MS------TSSPTLLCTPLMGTTVDQM
PgSDH3_2 121 GEDKAAQAMIPQYQSYPTGYSTTMASVPFTISDLQTSGSGFRSSPTLLCTPLMGTTVDLM
PgSDH3a_1 1 ---------------------------------MS------TSSRTLLCTPLMGTTVDQM


Arabidopsis_SDH 109 VIETSKAHELGADLVEIRLDWLKDFNPLEDLKTIIKKSPLPTLFTYRPKWEGGQYEGDEN
PgSDH1 26 AIDMDRAKASGADLVEIRLDCLKTFSPNEDLKTIMKASPLPTLITYRPKWEGGQYEGDEN
PgSDH3_1 38 LIEMRKAKEIGADVVEVRLDCLRKFNPFQDLEILIKRSPLPTLVTYRPFWEGGQYDGDDN
PgSDH4 15 LSLVRSAKDQGADLVELRLDFLKSFSPRQDLETLIQQSSLPTLVTFRPKWEGGQYEGDEK
PgSDH3a_2 22 LAEMRKAKEIGADVLEIRLDCLRKFNPHQDLEILIKRSPLPTLITYRPLREGGQYDGDEN
PgSDH3_2 181 LIEMGKAKEIGADLLEIRLDCLRAFNPRQDLDILIKQ----------PVWEGGQYEGDEN
PgSDH3a_1 22 LTEMRKAKEIGADVVEVRLDCLRKFNPHQDLEILIKRSPLPTLITYRPLREGGQYDGDEN


Arabidopsis_SDH 169 ERRDVLRLAMELGADYIDVELQVASEFIKSIDGKKPGKFKVIVSSHNYQNTPSVEDLDGL
PgSDH1 86 KRLDVLRLAVELGADYVDVELQVAREFNDSISGKKPQNCKVIVSSHNYESTPSIEDLGNL
PgSDH3_1 98 KRQDALRLAMHLGASYVDIELEVAYEFINSIHGKKPDNFKVIVSSHNFHNTPSSEAIGNL
PgSDH4 75 ARLDALRLAVELGSAYVDVELKVANEFFNSVKGKKPEKVKVIVSSHNYEKTPSSEELGNL
PgSDH3a_2 82 RRQDALRLAMHLGASYVDIELEVAYEFINSIRVKKPENFKVIVSSHNFHGTPSSEAIGKL
PgSDH3_2 231 KRQDALRLAMQLGANYVDVELEVAHDFNNSIQGKKPDSFKVIVSSHNFHNTPSAEAIGNL
PgSDH3a_1 82 KRQDALRLAMHLGASYVDIELEVAYEFINSIRVKKPENFKVIVSSHNFHETPSSEAIGKL


Arabidopsis_SDH 229 VARIQQTGADIVKIATTAVDIADVARMFHITSK--AQVPTIGLVMGERGLMSRILCSKFG
PgSDH1 146 VARIQAAGADIVKIATTALDITDVARIFQTIVHSQYQVPIIGLVMGERGLISRVLCAKYG
PgSDH3_1 158 VARIQASGADIVKVATTALDITDCARVFQIMVHSQ--IPTIGIVMGERGLISRLLSPKFG
PgSDH4 135 VARIQATGADIVKIATTATDITDNARIFQVLAHSQ--VPTIAIAMADRGYISRILSGKYG
PgSDH3a_2 142 VVMIQASGADIVKVATTALDITDCARVFQMTVQSQ--IPTIGIAMGERGLISRILSPKFG
PgSDH3_2 291 VARIQATGADIAKIATTALDITDCARIFQITVHSE--IPTIGIVMGERGLISRILSPKFG
PgSDH3a_1 142 VAMIQASGADIVKVATTALDITDCARVFQMMVQSQ--IPTIGISMGERGLISRILSPKFG


Arabidopsis_SDH 287 GYLTFGTLDSSKVSAPGQPTIKDLLDLYNFRRIGPDTKVYGIIGKPVSHSKSPIVHNQAF PgSDH1 206 GYLTFGTLDSGVVSAPGQPTVKDLLDLYNFRSIRHDTKVFGIIGKPVGHSKSPFLYNEAF
PgSDH3_1 216 AYLTYGALDTGAISAPGQPLAKDLLDLYNFRLIRPDTKVYGIIGKPVGHSKSPLLFNAAF
PgSDH4 193 AFLTFGTLEAGVESAPGQPTLKDLLDLYNFRQIGSDTKVHGVIGNPIGHSKSPHLYNSAF
PgSDH3a_2 200 AYLTYGALEASAISAPGQPLAKDLWDLYNFRLIRPDTKVYGVISKPVGHSKSPLLFNAAF
PgSDH3_2 349 GYLTYGALEAGAISAPGQPTAKDLLELYNFRLIRPDTKVYGIIGKPVGHSKSPLLFNAAF
PgSDH3a_1 200 AYLTYGALEASAISAPGQPLAKDLLDLYNFRLIRPDTKVYGVISKPVGHSKSPLLFNAAF

338

336

304

291

279

271

269

241

214

155

128

126

124

381

385

Arabidopsis_SDH 347 KSVDFNGVYVHLLVDNLVSFLQAYSSSDFA-GFSCTIPHKEAALQCCDEVDPLAKSIGAV
PgSDH1 266 KSAGFNGVYLHLLVDGVANFLQTYSSTDFT-GFSCTIPHKEEAVKCCDEVDPVAKSIGAV
PgSDH3_1 276 KSVGLNAVYVHLLVDDVEKFFEAYSAVDFVAGCSCTIPHKEVAVKCMGEIDPIAKKIGAI
PgSDH4 253 KSVNFNGIYLPLLVDNVANFISTFSSPDFV-GYSYTIPHKEAGLKCCDEVDPIAQAIGAI
PgSDH3a_2 260 KSAGLNAVYLHLLVDDVKKFFETYSAVDFVAGCSCGIPHKEVALKCMDEIDPIAKKIGAI
PgSDH3_2 409 KSVGLNAVYLHFLVDDVEKFFKTYSAVDFASGCSCTIPHKEAALKCMDEIDPIAKKIGAI
PgSDH3a_1 260 KSAGLNAVYVHLLVDDVKKFFETYSAADFVAGCSCGIPHKEVALKCMDEIDPIAKKIGAI

423

422

406

407

Arabidopsis_SDH 406 NTILRRKSDGKLLGYNTDCIGSISAIEDGLRSSGDPSSVPSSSSPLASKTVVVIGAGGAG
PgSDH1 325 NCIIRRASDGKLLGYNTDYVGAISAIEDGLQGSH--GASNSTSSPLYNKLFIVIGAGGAG
PgSDH3_1 336 NNIVRR-PDGTLTAFNTDYIGAISAIEDGLRELN--GATPGVGSPLAGKLFVVLGAGGAG
PgSDH4 312 SCMTRR-PDGSMMGYNVDYLGAIAAIEEALRARE--GT-NGAASPLAGKLFVVIGAGGAG
PgSDH3a_2 320 NNIMRR-PDGTLTAFNTDYIGAISAIEDGLRELN--GATPAVGSPLAGKLFVVLGAGGAG
PgSDH3_2 469 NNIVRR-PDGTLTAFNTDYIGAITAIEDGVRELN--GATPSAVSPLAGKLFVVLGAGGAG
PgSDH3a_1 320 NNIVRR-PDGTLAAFNTDYIGAISAIEDGLRELN--GATPAVGSPLAGKLFVVLGAGGAG

Arabidopsis_SDH 466 KALAYGAKEKGAKVVIANRTYERALELAEAIGGKALSLTDLDNYHPEDGMVLANTTSMGM
PgSDH1 383 KALAYGAKEKGARVVIANRTFERAKELADTIGGDALSLADLENFHPEDGMILANTTSIGM
PgSDH3_1 393 KSLAYGAAQKGARVVVANRTLERAKELADKVGGQAMTLDEVASFHPEDRMVLANTTSVGM
PgSDH4 368 KALAYGGYEKGARVMVANRTYDKAKELASKVGGKAITLEELENFHPEEGMILANTTSVGM
PgSDH3a_2 377 KALAYGAAQKGARIVVANRTLERAKELAGNVGGQAMTLDEVASFHPEDEMVLANATSVGM
PgSDH3_2 526 KSLAYGAAQKGARVVVANRTFERAKELAEKVGGKALTLEEVQDFHPEEGMILANTTSVGM
PgSDH3a_1 377 KALAHGAAQKGARIVVANRPLEQAKELADKVGGQAMTLDEVASFHPEDEMVLANATSVGM


Arabidopsis_SDH 526 QPNVEETPISKDALKHYALVFDAVYTPRITRLLREAEESGAITVSGSEMFVRQAYEQFEI
PgSDH1 443 QPKVDETPVPKQALKHYALVFDAVYTPKITRLLREAEECGATTVSGIEMFIGQAYGQYER
PgSDH3_1 453 KPNADGTPIPKLALRHYCLVFDAIYTPKDTRLLREARESGAIIVYGTEMLIRQGFEQYKN
PgSDH4 428 KPNIDRTPLAKKALSHYSLVFDAIYTPKLTRLLREAQECGATPVYGTEMFINQAFVQFER
PgSDH3a_2 437 TPKIDQTPIPKQALKNYCLVFDAVYTPKDTRLLRETRESGAFIVYGTEMLIRQGFEQYKN
PgSDH3_2 586 KPKIDETPLAQHALKHYSVVFDAIYTPKDTRLLKEARETGAVIVYGTEMLIRQGFEQYKN
PgSDH3a_1 437 TPKIDQTPIPKQALKNYCLVFDAVYTPKDTRLLREARESGAFIVYGTEMLIRQGFEQYKN


Arabidopsis_SDH 586 FTGLP-------------------------------------------------------
PgSDH1 503 YTGLP-------------------------------------------------------
PgSDH3_1 513 FTGLQ-------------------------------------------------------
PgSDH4 488 FTGMPERSIAASAVSCSQVEYLCSDRAVYVDICLFSCRLLVELVKVLSACGSFGRGVEFL
PgSDH3a_2 497 FMGLP-------------------------------------------------------
PgSDH3_2 646 FTGLP-------------------------------------------------------
PgSDH3a_1 497 FTGLP-------------------------------------------------------


.

582

578

550

**Additional File C.** Multiple sequence alignment of DQD/SDH proteins from *Vitis vinifera* and pomegranate. Six pomegranate and three *V. vinifera* DQD/SDH Proteins were aligned by T-Coffee.

PgSDH1 1 ------------------------------------------------------------
PgSDH3_1 1 MG----------------------------------------------------------
PgSDH4 1 ------------------------------------------------------------
PgSDH3a_2 1 MS----------------------------------------------------------
PgSDH3_2 1 MGREVSESCVESLLIEMVSSYCNRFYADKPELAARRIEAIGYQVGHQLSERYTMERPRFI
PgSDH3a_1 1 MS----------------------------------------------------------
VvSDH4 1 ------------------------------------------------------------
VvSDH3 1 M-----------------------------------------------------------
VvSDH2 1 ------------------------------------------------------------


PgSDH1 1 ------------------------------------------------------------
PgSDH3_1 3 ------------------------------------------------------------
PgSDH4 1 ------------------------------------------------------------
PgSDH3a_2 3 ------------------------------------------------------------
PgSDH3_2 61 DHLEAIKFICKDFWSEIFKKQIDNLKTNHRGTFVLQDNRFRWLSRMSIESSPETTGSSQD
PgSDH3a_1 3 ------------------------------------------------------------
VvSDH4 1 ------------------------------------------------------------
VvSDH3 2 ------------------------------------------------------------
VvSDH2 1 ------------------------------------------------------------


PgSDH1 1 -----------------------------------MEAAAMRQNSTLICVPIMGDSIDKM
PgSDH3_1 3 -------------------------SLSLAVSDIQTSTSGMRGSPTLLCTPLMGTTVDQM
PgSDH4 1 ----------------------------------------------MICAPLMADSVDQM
PgSDH3a_2 3 -------------------------------------TS----SPTLLCTPLMGTTVDQM
PgSDH3_2 121 GEDKAAQAMIPQYQSYPTGYSTTMASVPFTISDLQTSGSGFRSSPTLLCTPLMGTTVDLM
PgSDH3a_1 3 -------------------------------------TS----SRTLLCTPLMGTTVDQM
VvSDH4 1 ---------------------MT-LSSVPLATSDIQIPEGARRNSTLICVPIMADSVDQM
VvSDH3 2 ------------------------GSLPFTVSDLQTSVSGVRSNPTLLCTPLMGTTVEQM
VvSDH2 1 -----------------------------------MDDVGVLKKETMICTPLMGQSVEQM


PgSDH1 26 AIDMDRAKASGADLVEIRLDCLKTFSPNEDLKTIMKASPLPTLITYRPKWEGGQYEGDEN
PgSDH3_1 38 LIEMRKAKEIGADVVEVRLDCLRKFNPFQDLEILIKRSPLPTLVTYRPFWEGGQYDGDDN
PgSDH4 15 LSLVRSAKDQGADLVELRLDFLKSFSPRQDLETLIQQSSLPTLVTFRPKWEGGQYEGDEK
PgSDH3a_2 22 LAEMRKAKEIGADVLEIRLDCLRKFNPHQDLEILIKRSPLPTLITYRPLREGGQYDGDEN
PgSDH3_2 181 LIEMGKAKEIGADLLEIRLDCLRAFNPRQDLDILIK----------QPVWEGGQYEGDEN
PgSDH3a_1 22 LTEMRKAKEIGADVVEVRLDCLRKFNPHQDLEILIKRSPLPTLITYRPLREGGQYDGDEN
VvSDH4 39 LGQIRKAKEVGGDLVEIRLDYLKNFSPRQDLQFLVKQSPLPTLVTYRPTWEGGQYDGDEG
VvSDH3 38 LTEMRKAKEIGADIVEIRLDCLRNFSPAQDLQILIKQSPLPTLVTYRPIWEGGQYEGDEN
VvSDH2 26 VRDMHKAKVEGADLVEVRLDYINNFHPQQDLEIILRNKPLPVMIVYRPKWEGGQYEGDEH


PgSDH1 86 KRLDVLRLAVELGADYVDVELQVAREFNDSISGKKPQNCKVIVSSHNYESTPSIEDLGNL
PgSDH3_1 98 KRQDALRLAMHLGASYVDIELEVAYEFINSIHGKKPDNFKVIVSSHNFHNTPSSEAIGNL
PgSDH4 75 ARLDALRLAVELGSAYVDVELKVANEFFNSVKGKKPEKVKVIVSSHNYEKTPSSEELGNL
PgSDH3a_2 82 RRQDALRLAMHLGASYVDIELEVAYEFINSIRVKKPENFKVIVSSHNFHGTPSSEAIGKL
PgSDH3_2 231 KRQDALRLAMQLGANYVDVELEVAHDFNNSIQGKKPDSFKVIVSSHNFHNTPSAEAIGNL
PgSDH3a_1 82 KRQDALRLAMHLGASYVDIELEVAYEFINSIRVKKPENFKVIVSSHNFHETPSSEAIGKL
VvSDH4 99 KRLDALRLAIELGADYIDVELQVAPEFINSIQGKTSGKVKIIVSSHNYQNTPSAEELGNL
VvSDH3 98 KRQDALRLAMELGASYIDVELEVAHEFNNSIYGKKPQNFKVIVSSHNFHNTPSTEAIGNL
VvSDH2 86 SRLEALHLAEKLGADYIDFELKVASDFLGKQKMDQHSSSRTIVSCYVDGVTPPIEDLICR


PgSDH1 146 VARIQAAGADIVKIATTALDITDVARIFQTIVHSQYQVPIIGLVMGERGLISRVLCAKYG
PgSDH3_1 158 VARIQASGADIVKVATTALDITDCARVFQIMVHSQ--IPTIGIVMGERGLISRLLSPKFG
PgSDH4 135 VARIQATGADIVKIATTATDITDNARIFQVLAHSQ--VPTIAIAMADRGYISRILSGKYG
PgSDH3a_2 142 VVMIQASGADIVKVATTALDITDCARVFQMTVQSQ--IPTIGIAMGERGLISRILSPKFG
PgSDH3_2 291 VARIQATGADIAKIATTALDITDCARIFQITVHSE--IPTIGIVMGERGLISRILSPKFG
PgSDH3a_1 142 VAMIQASGADIVKVATTALDITDCARVFQMMVQSQ--IPTIGISMGERGLISRILSPKFG
VvSDH4 159 VARIQATGADIVKIATTALDITDCARIFQVLAHSQ--VPTIGIAMAERGLISRILSAKFG
VvSDH3 158 VARIQASGADIVKIATTALDITDVARVLQVTVHSQ--VPTIAIVMGERGLISRLLSPKFG
VvSDH2 146 VALLQSTGADMIKLVINATNITEITKIFHLLSHCQ--MPLIAYSIGDRGFMSQILCRKFG
PgSDH1 206 GYLTFGTLDSGVVSAPGQPTVKDLLDLYNFRSIRHDTKVFGIIGKPVGHSKSPFLYNEAF
PgSDH3_1 216 AYLTYGALDTGAISAPGQPLAKDLLDLYNFRLIRPDTKVYGIIGKPVGHSKSPLLFNAAF
PgSDH4 193 AFLTFGTLEAGVESAPGQPTLKDLLDLYNFRQIGSDTKVHGVIGNPIGHSKSPHLYNSAF
PgSDH3a_2 200 AYLTYGALEASAISAPGQPLAKDLWDLYNFRLIRPDTKVYGVISKPVGHSKSPLLFNAAF
PgSDH3_2 349 GYLTYGALEAGAISAPGQPTAKDLLELYNFRLIRPDTKVYGIIGKPVGHSKSPLLFNAAF
PgSDH3a_1 200 AYLTYGALEASAISAPGQPLAKDLLDLYNFRLIRPDTKVYGVISKPVGHSKSPLLFNAAF
VvSDH4 217 SYLTFGSLEAGVVSAPGQPTVKDLLDLYNFRQIGPDTKVHGVIGKPIGHSKSPHLYNSAF
VvSDH3 216 GYLTYGALEAGAISAPGQPTAKDLLDLYNFRLVKPDTKVYGIIGKPVGHSKSPLLFNAAF
VvSDH2 204 GFLVYGSMEGSPVA--GLPTLESLREAYKVQYINKDTKVFGLISKPVGHSKGPILHNPAF


PgSDH1 266 KSAGFNGVYLHLLVDGVANFLQTYSSTDFTG-FSCTIPHKEEAVKCCDEVDPVAKSIGAV
PgSDH3_1 276 KSVGLNAVYVHLLVDDVEKFFEAYSAVDFVAGCSCTIPHKEVAVKCMGEIDPIAKKIGAI
PgSDH4 253 KSVNFNGIYLPLLVDNVANFISTFSSPDFVG-YSYTIPHKEAGLKCCDEVDPIAQAIGAI
PgSDH3a_2 260 KSAGLNAVYLHLLVDDVKKFFETYSAVDFVAGCSCGIPHKEVALKCMDEIDPIAKKIGAI
PgSDH3_2 409 KSVGLNAVYLHFLVDDVEKFFKTYSAVDFASGCSCTIPHKEAALKCMDEIDPIAKKIGAI
PgSDH3a_1 260 KSAGLNAVYVHLLVDDVKKFFETYSAADFVAGCSCGIPHKEVALKCMDEIDPIAKKIGAI
VvSDH4 277 KSVGFNGIYLPLLVDSVKNFLATYSSPDFVG-YSYTIPHKEDGLRCCDEIDPIAQAIGAI
VvSDH3 276 KKVGLNAVYVHLLVDDVEKFFNTYSAPDFISGCSCTIPHKEVAIKCMDTIDPIARKIGAI
VvSDH2 262 RHVNYNGIYVPMLVDDLKEFFSIYSSPDFA-GFSVGIPYKEAVTGFCDELHPLAQSIGAV


PgSDH1 325 NCIIRRASDGKLLGYNTDYVGAISAIEDGLQG--SH-GASNSTSSPLYNKLFIVIGAGGA
PgSDH3_1 336 NNIVRRP-DGTLTAFNTDYIGAISAIEDGLRE--LN-GATPGVGSPLAGKLFVVLGAGGA
PgSDH4 312 SCMTRRP-DGSMMGYNVDYLGAIAAIEEALRA--RE-G-TNGAASPLAGKLFVVIGAGGA
PgSDH3a_2 320 NNIMRRP-DGTLTAFNTDYIGAISAIEDGLRE--LN-GATPAVGSPLAGKLFVVLGAGGA
PgSDH3_2 469 NNIVRRP-DGTLTAFNTDYIGAITAIEDGVRE--LN-GATPSAVSPLAGKLFVVLGAGGA
PgSDH3a_1 320 NNIVRRP-DGTLAAFNTDYIGAISAIEDGLRE--LN-GATPAVGSPLAGKLFVVLGAGGA
VvSDH4 336 SCMIRRPADGKLMGYNVDYLGAIAAIEEGLRA--SN-G-TTSVGSPLAGKLFVVIGAGGA
VvSDH3 336 NNIVRKP-DGKLTAFNTDYIGAIEAIEDGLRE--SN-GSSPAVGSPLAGKLFVVLGAGGA
VvSDH2 321 NTIMRRPSDGKLIGYNTDCEASITAIEDALRERGLPNGEAP-LNSPLTGKQFVLVGAGGA


PgSDH1 382 GKALAYGAKEKGARVVIANRTFERAKELADTIGGDALSLADLENFHPEDGMILANTTSIG
PgSDH3_1 392 GKSLAYGAAQKGARVVVANRTLERAKELADKVGGQAMTLDEVASFHPEDRMVLANTTSVG
PgSDH4 367 GKALAYGGYEKGARVMVANRTYDKAKELASKVGGKAITLEELENFHPEEGMILANTTSVG
PgSDH3a_2 376 GKALAYGAAQKGARIVVANRTLERAKELAGNVGGQAMTLDEVASFHPEDEMVLANATSVG
PgSDH3_2 525 GKSLAYGAAQKGARVVVANRTFERAKELAEKVGGKALTLEEVQDFHPEEGMILANTTSVG
PgSDH3a_1 376 GKALAHGAAQKGARIVVANRPLEQAKELADKVGGQAMTLDEVASFHPEDEMVLANATSVG
VvSDH4 392 GKALAYGGKEKGARVVVANRTFEKAKELASKVGGEAMTLAELENFHPEDGMILANTTSVG
VvSDH3 392 GKSLAYGAKEKGARVVVANRTFERAKDLADKVGGQALTLAEIENFHPEEGMILANTTSVG
VvSDH2 380 GRALAFGARSRGAQLVIFDLDFDRANSLAHAVSGEVKLYEDVANFQPEKGAILANATPVG


PgSDH1 442 MQPKVDETPVPKQALKHYALVFDAVYTPKITRLLREAEECGATTVSGIEMFIGQAYGQYE
PgSDH3_1 452 MKPNADGTPIPKLALRHYCLVFDAIYTPKDTRLLREARESGAIIVYGTEMLIRQGFEQYK
PgSDH4 427 MKPNIDRTPLAKKALSHYSLVFDAIYTPKLTRLLREAQECGATPVYGTEMFINQAFVQFE
PgSDH3a_2 436 MTPKIDQTPIPKQALKNYCLVFDAVYTPKDTRLLRETRESGAFIVYGTEMLIRQGFEQYK
PgSDH3_2 585 MKPKIDETPLAQHALKHYSVVFDAIYTPKDTRLLKEARETGAVIVYGTEMLIRQGFEQYK
PgSDH3a_1 436 MTPKIDQTPIPKQALKNYCLVFDAVYTPKDTRLLREARESGAFIVYGTEMLIRQGFEQYK
VvSDH4 452 MKPNIDNTPLSKKALSRYSLVFDAIYTPKLTRLLREAQESGAIIVYGTEMFINQAFVQFE
VvSDH3 452 MKPKINDTPIPKHALKHYSLVFDAIYTPKDTRLLREAKESGKIIVYGTEMLIRQGFEQYK
VvSDH2 440 MHPNTDRIPVAEETLSDYQLVFDSVYTPRKTRLLKEAEAAGAIIVSGVEMFLRQAIGQFN


PgSDH1 502 RYTGLPAP------------------------------KELFRKLMST------------
PgSDH3_1 512 NFTGLQAP------------------------------EELFRTLMEKH-----------
PgSDH4 487 RFTGMPERSIAASAVSCSQVEYLCSDRAVYVDICLFSCRLLVELVKVLSACGSFGRGVEF
PgSDH3a_2 496 NFMGLPAP------------------------------EELFRMVMEKH-----------
PgSDH3_2 645 NFTGLPAP------------------------------EELFRQLMEKH-----------
PgSDH3a_1 496 NFTGLPAP------------------------------EELFRMVMMKH-----------
VvSDH4 512 RFTGLPAP------------------------------KELIREVLVRN-----------
VvSDH3 512 NFTGLPAP------------------------------EELFRELMSKH-----------
VvSDH2 500 LFTGGEAP------------------------------EEFMREIILSK-----------
PgSDH1 520 ------------------------------------------------------------
PgSDH3_1 531 ------------------------------------------------------------
PgSDH4 547 LLGIDYGEVSRSVDIAGGLGPEMKLACSTDWGQKQVACIREEEESMDEAEADGEAFTELQ
PgSDH3a_2 515 ------------------------------------------------------------
PgSDH3_2 664 ------------------------------------------------------------
PgSDH3a_1 515 ------------------------------------------------------------
VvSDH4 531 ------------------------------------------------------------
VvSDH3 531 ------------------------------------------------------------
VvSDH2 519 ------------------------------------------------------------


PgSDH1 520 ------------------------------------------------------------
PgSDH3_1 531 ------------------------------------------------------------
PgSDH4 607 PVEQEDKKEAETPVEPSTVLQTIEQAETPSASNKYLVAKQGSKEETETPPETFPELQIVE
PgSDH3a_2 515 ------------------------------------------------------------
PgSDH3_2 664 ------------------------------------------------------------
PgSDH3a_1 515 ------------------------------------------------------------
VvSDH4 531 ------------------------------------------------------------
VvSDH3 531 ------------------------------------------------------------
VvSDH2 519 ------------------------------------------------------------


PgSDH1 520 ------------------------------------------------------------
PgSDH3_1 531 ------------------------------------------------------------
PgSDH4 667 QETKNECEMPSEASVRSRPPEKKAANSLKARAKMVNKSAAKSFLNAKNGKAALKVQKKIV
PgSDH3a_2 515 ------------------------------------------------------------
PgSDH3_2 664 ------------------------------------------------------------
PgSDH3a_1 515 ------------------------------------------------------------
VvSDH4 531 ------------------------------------------------------------
VvSDH3 531 ------------------------------------------------------------
VvSDH2 519 ------------------------------------------------------------


PgSDH1 520 ------------------------------------------------------------
PgSDH3_1 531 ------------------------------------------------------------
PgSDH4 727 KKNKGAPLVINEGSKDGGETGGAKEEYVEQENKKEAETPTEVSTMFHSSEKGTGKEAEMP
PgSDH3a_2 515 ------------------------------------------------------------
PgSDH3_2 664 ------------------------------------------------------------
PgSDH3a_1 515 ------------------------------------------------------------
VvSDH4 531 ------------------------------------------------------------
VvSDH3 531 ------------------------------------------------------------
VvSDH2 519 ------------------------------------------------------------


PgSDH1 520 ------------------------------------------------------------
PgSDH3_1 531 ------------------------------------------------------------
PgSDH4 787 ATSVQAEAISPQKRTPNSLKVGAKIVKKVSPNKLWNTAGRKAALNKVQKKIVKNKSLVLN
PgSDH3a_2 515 ------------------------------------------------------------
PgSDH3_2 664 ------------------------------------------------------------
PgSDH3a_1 515 ------------------------------------------------------------
VvSDH4 531 ------------------------------------------------------------
VvSDH3 531 ------------------------------------------------------------
VvSDH2 519 ------------------------------------------------------------


PgSDH1 520 ------------------------------------------------------------
PgSDH3_1 531 ------------------------------------------------------------
PgSDH4 847 SSGKNSGETANDNTLTVSVTDVKDNPGNKRKDTSHNDDKNGREPALGDINDSGKQIVQGK
PgSDH3a_2 515 ------------------------------------------------------------
PgSDH3_2 664 ------------------------------------------------------------
PgSDH3a_1 515 ------------------------------------------------------------
VvSDH4 531 ------------------------------------------------------------
VvSDH3 531 ------------------------------------------------------------
VvSDH2 519 ------------------------------------------------------------
PgSDH1 520 ------------------------------------------------------------
PgSDH3_1 531 ------------------------------------------------------------
PgSDH4 907 GGKTKRRQRKHENNQGRSSNDGGENPKKNERLEREEMDQGKKGKEEKEKIGGLILLCSTK
PgSDH3a_2 515 ------------------------------------------------------------
PgSDH3_2 664 ------------------------------------------------------------
PgSDH3a_1 515 ------------------------------------------------------------
VvSDH4 531 ------------------------------------------------------------
VvSDH3 531 ------------------------------------------------------------
VvSDH2 519 ------------------------------------------------------------


PgSDH1 520 ------------------------------------------------------------
PgSDH3_1 531 ------------------------------------------------------------
PgSDH4 967 TKPDCLNYRVMGVPESQKDMVLAVKPGLKLFLYDFDLKLMYGIYKASSAGGKKLEPRAFG
PgSDH3a_2 515 ------------------------------------------------------------
PgSDH3_2 664 ------------------------------------------------------------
PgSDH3a_1 515 ------------------------------------------------------------
VvSDH4 531 ------------------------------------------------------------
VvSDH3 531 ------------------------------------------------------------
VvSDH2 519 ------------------------------------------------------------


PgSDH1 520 ------------------------------------------------------------
PgSDH3_1 531 ------------------------------------------------------------
PgSDH4 1027 GAFPYQVRFRTHIDCFPLPETIFKKAMMENFITKHKFKTELTFQQVRKLTELFRPVEIHP
PgSDH3a_2 515 ------------------------------------------------------------
PgSDH3_2 664 ------------------------------------------------------------
PgSDH3a_1 515 ------------------------------------------------------------
VvSDH4 531 ------------------------------------------------------------
VvSDH3 531 ------------------------------------------------------------
VvSDH2 519 ------------------------------------------------------------


PgSDH1 520 ------------------------------------------------------------
PgSDH3_1 531 ------------------------------------------------------------
PgSDH4 1087 SVQPRPDKFPPRSARKERARDRDARLSMSESQHHSQRETVRTDPYAGRDERRYPLLAREG
PgSDH3a_2 515 ------------------------------------------------------------
PgSDH3_2 664 ------------------------------------------------------------
PgSDH3a_1 515 ------------------------------------------------------------
VvSDH4 531 ------------------------------------------------------------
VvSDH3 531 ------------------------------------------------------------
VvSDH2 519 ------------------------------------------------------------


PgSDH1 520 ------------------------------------------------------------
PgSDH3_1 531 ------------------------------------------------------------
PgSDH4 1147 DRLTANREVRAEPPREMFLTEADYRAYGLRGWRRYSDLPSRAAPPLDPYWEDYEKRRQPD
PgSDH3a_2 515 ------------------------------------------------------------
PgSDH3_2 664 ------------------------------------------------------------
PgSDH3a_1 515 ------------------------------------------------------------
VvSDH4 531 ------------------------------------------------------------
VvSDH3 531 ------------------------------------------------------------
VvSDH2 519 ------------------------------------------------------------


PgSDH1 520 ------------------------------------------------------------
PgSDH3_1 531 ------------------------------------------------------------
PgSDH4 1207 LVYRDAVPARREYVYANPSYSDYRDCQTYSSYDARREYPIVHRYPDYQRRAAVSSSEAYP
PgSDH3a_2 515 ------------------------------------------------------------
PgSDH3_2 664 ------------------------------------------------------------
PgSDH3a_1 515 ------------------------------------------------------------
VvSDH4 531 ------------------------------------------------------------
VvSDH3 531 ------------------------------------------------------------
VvSDH2 519 ------------------------------------------------------------
PgSDH1 520 ------------------------------------------------K
PgSDH3_1 531 ------------------------------------------------A
PgSDH4 1267 TESNRDLRRRERDPVDRIYTEYDMRRYHIADPDLAALPVSSRYSFAGPR
PgSDH3a_2 515 ------------------------------------------------A
PgSDH3_2 664 ------------------------------------------------A
PgSDH3a_1 515 ------------------------------------------------A
VvSDH4 531 ------------------------------------------------T
VvSDH3 531 ------------------------------------------------A
VvSDH2 519 ------------------------------------------------F

Additional file D. Alignment analysis between SDH proteins belonging to PgSDH3 and PgSDH3-a. Alignments were performed with T-Coffee

PgSDH3_1 1 ------------------------------------------------------------
PgSDH3_2 1 MGREVSESCVESLLIEMVSSYCNRFYADKPELAARRIEAIGYQVGHQLSERYTMERPRFI
PgSDH3a_1 1 ------------------------------------------------------------
PgSDH3a_2 1 ------------------------------------------------------------


PgSDH3_1 1 ------------------------------------------------------------
PgSDH3_2 61 DHLEAIKFICKDFWSEIFKKQIDNLKTNHRGTFVLQDNRFRWLSRMSIESSPETTGSSQD
PgSDH3a_1 1 ------------------------------------------------------------
PgSDH3a_2 1 ------------------------------------------------------------


PgSDH3_1 1 -----------------------MGSLSLAVSDIQTSTSGMRGSPTLLCTPLMGTTVDQM
PgSDH3_2 121 GEDKAAQAMIPQYQSYPTGYSTTMASVPFTISDLQTSGSGFRSSPTLLCTPLMGTTVDLM
PgSDH3a_1 1 -----------------------------------MS----TSSRTLLCTPLMGTTVDQM
PgSDH3a_2 1 -----------------------------------MS----TSSPTLLCTPLMGTTVDQM


PgSDH3_1 38 LIEMRKAKEIGADVVEVRLDCLRKFNPFQDLEILIKRSPLPTLVTYRPFWEGGQYDGDDN
PgSDH3_2 181 LIEMGKAKEIGADLLEIRLDCLRAFNPRQDLDILIK----------QPVWEGGQYEGDEN
PgSDH3a_1 22 LTEMRKAKEIGADVVEVRLDCLRKFNPHQDLEILIKRSPLPTLITYRPLREGGQYDGDEN
PgSDH3a_2 22 LAEMRKAKEIGADVLEIRLDCLRKFNPHQDLEILIKRSPLPTLITYRPLREGGQYDGDEN


PgSDH3_1 98 KRQDALRLAMHLGASYVDIELEVAYEFINSIHGKKPDNFKVIVSSHNFHNTPSSEAIGNL
PgSDH3_2 231 KRQDALRLAMQLGANYVDVELEVAHDFNNSIQGKKPDSFKVIVSSHNFHNTPSAEAIGNL
PgSDH3a_1 82 KRQDALRLAMHLGASYVDIELEVAYEFINSIRVKKPENFKVIVSSHNFHETPSSEAIGKL
PgSDH3a_2 82 RRQDALRLAMHLGASYVDIELEVAYEFINSIRVKKPENFKVIVSSHNFHGTPSSEAIGKL


PgSDH3_1 158 VARIQASGADIVKVATTALDITDCARVFQIMVHSQIPTIGIVMGERGLISRLLSPKFGAY
PgSDH3_2 291 VARIQATGADIAKIATTALDITDCARIFQITVHSEIPTIGIVMGERGLISRILSPKFGGY
PgSDH3a_1 142 VAMIQASGADIVKVATTALDITDCARVFQMMVQSQIPTIGISMGERGLISRILSPKFGAY
PgSDH3a_2 142 VVMIQASGADIVKVATTALDITDCARVFQMTVQSQIPTIGIAMGERGLISRILSPKFGAY


PgSDH3_1 218 LTYGALDTGAISAPGQPLAKDLLDLYNFRLIRPDTKVYGIIGKPVGHSKSPLLFNAAFKS
PgSDH3_2 351 LTYGALEAGAISAPGQPTAKDLLELYNFRLIRPDTKVYGIIGKPVGHSKSPLLFNAAFKS
PgSDH3a_1 202 LTYGALEASAISAPGQPLAKDLLDLYNFRLIRPDTKVYGVISKPVGHSKSPLLFNAAFKS
PgSDH3a_2 202 LTYGALEASAISAPGQPLAKDLWDLYNFRLIRPDTKVYGVISKPVGHSKSPLLFNAAFKS


PgSDH3_1 278 VGLNAVYVHLLVDDVEKFFEAYSAVDFVAGCSCTIPHKEVAVKCMGEIDPIAKKIGAINN
PgSDH3_2 411 VGLNAVYLHFLVDDVEKFFKTYSAVDFASGCSCTIPHKEAALKCMDEIDPIAKKIGAINN
PgSDH3a_1 262 AGLNAVYVHLLVDDVKKFFETYSAADFVAGCSCGIPHKEVALKCMDEIDPIAKKIGAINN
PgSDH3a_2 262 AGLNAVYLHLLVDDVKKFFETYSAVDFVAGCSCGIPHKEVALKCMDEIDPIAKKIGAINN


PgSDH3_1 338 IVRRPDGTLTAFNTDYIGAISAIEDGLRELNGATPGVGSPLAGKLFVVLGAGGAGKSLAY
PgSDH3_2 471 IVRRPDGTLTAFNTDYIGAITAIEDGVRELNGATPSAVSPLAGKLFVVLGAGGAGKSLAY
PgSDH3a_1 322 IVRRPDGTLAAFNTDYIGAISAIEDGLRELNGATPAVGSPLAGKLFVVLGAGGAGKALAH
PgSDH3a_2 322 IMRRPDGTLTAFNTDYIGAISAIEDGLRELNGATPAVGSPLAGKLFVVLGAGGAGKALAY


PgSDH3_1 398 GAAQKGARVVVANRTLERAKELADKVGGQAMTLDEVASFHPEDRMVLANTTSVGMKPNAD
PgSDH3_2 531 GAAQKGARVVVANRTFERAKELAEKVGGKALTLEEVQDFHPEEGMILANTTSVGMKPKID
PgSDH3a_1 382 GAAQKGARIVVANRPLEQAKELADKVGGQAMTLDEVASFHPEDEMVLANATSVGMTPKID
PgSDH3a_2 382 GAAQKGARIVVANRTLERAKELAGNVGGQAMTLDEVASFHPEDEMVLANATSVGMTPKID


PgSDH3_1 458 GTPIPKLALRHYCLVFDAIYTPKDTRLLREARESGAIIVYGTEMLIRQGFEQYKNFTGLQ
PgSDH3_2 591 ETPLAQHALKHYSVVFDAIYTPKDTRLLKEARETGAVIVYGTEMLIRQGFEQYKNFTGLP
PgSDH3a_1 442 QTPIPKQALKNYCLVFDAVYTPKDTRLLREARESGAFIVYGTEMLIRQGFEQYKNFTGLP
PgSDH3a_2 442 QTPIPKQALKNYCLVFDAVYTPKDTRLLRETRESGAFIVYGTEMLIRQGFEQYKNFMGLP


PgSDH3_1 518 APEELFRTLMEKHA
PgSDH3_2 651 APEELFRQLMEKHA
PgSDH3a_1 502 APEELFRMVMMKHA
PgSDH3a_2 502 APEELFRMVMEKHA

**Additional File E.** Blast analysis between PgSDH4 and VvSDH4, between VvSDH3, and PgSDH3-2 and between VvSDH1, and PgSDH1.

# Aligned_sequences: 2

# 1: VvSDH4

# 2: PgSDH4

# Matrix: EBLOSUM62

# Gap_penalty: 10.0

# Extend_penalty: 0.5

#

# Length: 493

# Identity: 405/493 (82.2%)

# Similarity: 447/493 (90.7%)

# Gaps: 1/493 ( 0.2%)

# Score: 2101.0

#

#

#=======================================

VvSDH4 25 LICVPIMADSVDQMLGQIRKAKEVGGDLVEIRLDYLKNFSPRQDLQFLVK 74

:||.|:|||||||||..:|.||:.|.||||:|||:||:|||||||:.|::

PgSDH4 1 MICAPLMADSVDQMLSLVRSAKDQGADLVELRLDFLKSFSPRQDLETLIQ 50

VvSDH4 75 QSPLPTLVTYRPTWEGGQYDGDEGKRLDALRLAIELGADYIDVELQVAPE 124

||.||||||:||.||||||:|||..||||||||:|||:.|:||||:||.|

PgSDH4 51 QSSLPTLVTFRPKWEGGQYEGDEKARLDALRLAVELGSAYVDVELKVANE 100

VvSDH4 125 FINSIQGKTSGKVKIIVSSHNYQNTPSAEELGNLVARIQATGADIVKIAT 174

|.||::||...|||:|||||||:.|||:||||||||||||||||||||||

PgSDH4 101 FFNSVKGKKPEKVKVIVSSHNYEKTPSSEELGNLVARIQATGADIVKIAT 150

VvSDH4 175 TALDITDCARIFQVLAHSQVPTIGIAMAERGLISRILSAKFGSYLTFGSL 224

||.||||.|||||||||||||||.||||:||.||||||.|:|::||||:|

PgSDH4 151 TATDITDNARIFQVLAHSQVPTIAIAMADRGYISRILSGKYGAFLTFGTL 200

VvSDH4 225 EAGVVSAPGQPTVKDLLDLYNFRQIGPDTKVHGVIGKPIGHSKSPHLYNS 274

||||.|||||||:|||||||||||||.|||||||||.|||||||||||||

PgSDH4 201 EAGVESAPGQPTLKDLLDLYNFRQIGSDTKVHGVIGNPIGHSKSPHLYNS 250

VvSDH4 275 AFKSVGFNGIYLPLLVDSVKNFLATYSSPDFVGYSYTIPHKEDGLRCCDE 324

|||||.|||||||||||:|.||::|:||||||||||||||||.||:||||

PgSDH4 251 AFKSVNFNGIYLPLLVDNVANFISTFSSPDFVGYSYTIPHKEAGLKCCDE 300

VvSDH4 325 IDPIAQAIGAISCMIRRPADGKLMGYNVDYLGAIAAIEEGLRASNGTTSV 374

:|||||||||||||.||| ||.:||||||||||||||||.|||..||...

PgSDH4 301 VDPIAQAIGAISCMTRRP-DGSMMGYNVDYLGAIAAIEEALRAREGTNGA 349

VvSDH4 375 GSPLAGKLFVVIGAGGAGKALAYGGKEKGARVVVANRTFEKAKELASKVG 424

.||||||||||||||||||||||||.||||||:|||||::||||||||||

PgSDH4 350 ASPLAGKLFVVIGAGGAGKALAYGGYEKGARVMVANRTYDKAKELASKVG 399

VvSDH4 425 GEAMTLAELENFHPEDGMILANTTSVGMKPNIDNTPLSKKALSRYSLVFD 474

|:|:||.||||||||:|||||||||||||||||.|||:|||||.||||||

PgSDH4 400 GKAITLEELENFHPEEGMILANTTSVGMKPNIDRTPLAKKALSHYSLVFD 449

VvSDH4 475 AIYTPKLTRLLREAQESGAIIVYGTEMFINQAFVQFERFTGLP 517

||||||||||||||||.||..||||||||||||||||||||:|

PgSDH4 450 AIYTPKLTRLLREAQECGATPVYGTEMFINQAFVQFERFTGMP 492

# Aligned_sequences: 2

# 1: VvSDH3

# 2: PgSDH3_2

# Matrix: EBLOSUM62

# Gap_penalty: 10.0

# Extend_penalty: 0.5

#

# Length: 531

# Identity: 441/531 (83.1%)

# Similarity: 488/531 (91.9%)

# Gaps: 10/531 ( 1.9%)

# Score: 2319.5

#

#

#=======================================

VvSDH3 1 MGSLPFTVSDLQTSVSGVRSNPTLLCTPLMGTTVEQMLTEMRKAKEIGAD 50

|.|:|||:||||||.||.||:|||||||||||||:.||.||.||||||||

PgSDH3_2 144 MASVPFTISDLQTSGSGFRSSPTLLCTPLMGTTVDLMLIEMGKAKEIGAD 193

VvSDH3 51 IVEIRLDCLRNFSPAQDLQILIKQSPLPTLVTYRPIWEGGQYEGDENKRQ 100

::||||||||.|:|.|||.||||| |:||||||||||||||

PgSDH3_2 194 LLEIRLDCLRAFNPRQDLDILIKQ----------PVWEGGQYEGDENKRQ 233

VvSDH3 101 DALRLAMELGASYIDVELEVAHEFNNSIYGKKPQNFKVIVSSHNFHNTPS 150

|||||||:|||:|:||||||||:|||||.||||.:|||||||||||||||

PgSDH3_2 234 DALRLAMQLGANYVDVELEVAHDFNNSIQGKKPDSFKVIVSSHNFHNTPS 283

VvSDH3 151 TEAIGNLVARIQASGADIVKIATTALDITDVARVLQVTVHSQVPTIAIVM 200

.||||||||||||:||||.|||||||||||.||:.|:||||::|||.|||

PgSDH3_2 284 AEAIGNLVARIQATGADIAKIATTALDITDCARIFQITVHSEIPTIGIVM 333

VvSDH3 201 GERGLISRLLSPKFGGYLTYGALEAGAISAPGQPTAKDLLDLYNFRLVKP 250

||||||||:|||||||||||||||||||||||||||||||:||||||::|

PgSDH3_2 334 GERGLISRILSPKFGGYLTYGALEAGAISAPGQPTAKDLLELYNFRLIRP 383

VvSDH3 251 DTKVYGIIGKPVGHSKSPLLFNAAFKKVGLNAVYVHLLVDDVEKFFNTYS 300

||||||||||||||||||||||||||.|||||||:|.|||||||||.|||

PgSDH3_2 384 DTKVYGIIGKPVGHSKSPLLFNAAFKSVGLNAVYLHFLVDDVEKFFKTYS 433

VvSDH3 301 APDFISGCSCTIPHKEVAIKCMDTIDPIARKIGAINNIVRKPDGKLTAFN 350

|.||.|||||||||||.|:||||.|||||:||||||||||:|||.|||||

PgSDH3_2 434 AVDFASGCSCTIPHKEAALKCMDEIDPIAKKIGAINNIVRRPDGTLTAFN 483

VvSDH3 351 TDYIGAIEAIEDGLRESNGSSPAVGSPLAGKLFVVLGAGGAGKSLAYGAK 400

|||||||.|||||:||.||::|:..||||||||||||||||||||||||.

PgSDH3_2 484 TDYIGAITAIEDGVRELNGATPSAVSPLAGKLFVVLGAGGAGKSLAYGAA 533

VvSDH3 401 EKGARVVVANRTFERAKDLADKVGGQALTLAEIENFHPEEGMILANTTSV 450

:||||||||||||||||:||:||||:||||.|:::|||||||||||||||

PgSDH3_2 534 QKGARVVVANRTFERAKELAEKVGGKALTLEEVQDFHPEEGMILANTTSV 583

VvSDH3 451 GMKPKINDTPIPKHALKHYSLVFDAIYTPKDTRLLREAKESGKIIVYGTE 500

||||||::||:.:|||||||:||||||||||||||:||:|:|.:||||||

PgSDH3_2 584 GMKPKIDETPLAQHALKHYSVVFDAIYTPKDTRLLKEARETGAVIVYGTE 633

VvSDH3 501 MLIRQGFEQYKNFTGLPAPEELFRELMSKHA 531

||||||||||||||||||||||||:||.|||

PgSDH3_2 634 MLIRQGFEQYKNFTGLPAPEELFRQLMEKHA 664

#=======================================

#

# Aligned_sequences: 2

# 1: VvSDH1

# 2: PgSDH1

# Matrix: EBLOSUM62

# Gap_penalty: 10.0

# Extend_penalty: 0.5

#

# Length: 518

# Identity: 396/518 (76.4%)

# Similarity: 455/518 (87.8%)

# Gaps: 2/518 ( 0.4%)

# Score: 2105.5

#

#

#=======================================

VvSDH1 1 MESGGMSKNSTLICVPIMGETIEKMVVDMSKAKTSGADLVEVRLDTLKRF 50

||:..|.:|||||||||||::|:||.:||.:||.|||||||:|||.||.|

PgSDH1 1 MEAAAMRQNSTLICVPIMGDSIDKMAIDMDRAKASGADLVEIRLDCLKTF 50

VvSDH1 51 NPRQDLEVLIRKCPLPTLFTYRPKWEGGQYEGDENSRRDALRLAMELGAD 100

:|.:||:.:::..|||||.||||||||||||||||.|.|.||||:|||||

PgSDH1 51 SPNEDLKTIMKASPLPTLITYRPKWEGGQYEGDENKRLDVLRLAVELGAD 100

VvSDH1 101 YVDIELKVAHEFINSIHGRKPEKFKVIVSSHNYQNTPSVEDLGNLVVSIQ 150

|||:||:||.||.:||.|:||:..|||||||||::|||:|||||||..||

PgSDH1 101 YVDVELQVAREFNDSISGKKPQNCKVIVSSHNYESTPSIEDLGNLVARIQ 150

VvSDH1 151 ATGADIVKIATTALEITDVARIFQITVHS--QVPVIGLVMGERGLISRIL 198

|.||||||||||||:|||||||||..||| |||:|||||||||||||:|

PgSDH1 151 AAGADIVKIATTALDITDVARIFQTIVHSQYQVPIIGLVMGERGLISRVL 200

VvSDH1 199 CPKFSGYLTFGSLEPGIVSAPGQPTIKDLLNLYNFRQLGPDTKVFGVIGK 248

|.|:.||||||:|:.|:||||||||:||||:|||||.:..||||||:|||

PgSDH1 201 CAKYGGYLTFGTLDSGVVSAPGQPTVKDLLDLYNFRSIRHDTKVFGIIGK 250

VvSDH1 249 PVSHSKSPHLYNEAFKSVGFNGVYVHLLVDDIAKFFHTYSAADFAGFSCT 298

||.|||||.||||||||.||||||:|||||.:|.|..|||:.||.|||||

PgSDH1 251 PVGHSKSPFLYNEAFKSAGFNGVYLHLLVDGVANFLQTYSSTDFTGFSCT 300

VvSDH1 299 IPHKEAALKCCDEVSPVAKSIGAVNCIIRRPSDGKLFGYNTDYVGAISAI 348

|||||.|:||||||.|||||||||||||||.|||||.|||||||||||||

PgSDH1 301 IPHKEEAVKCCDEVDPVAKSIGAVNCIIRRASDGKLLGYNTDYVGAISAI 350

VvSDH1 349 EDGLRDLHKISSTSGSPLAGKLFVVIGAGGAGKALAYGAKEKGARVVIAN 398

||||:..|..|:::.|||..|||:||||||||||||||||||||||||||

PgSDH1 351 EDGLQGSHGASNSTSSPLYNKLFIVIGAGGAGKALAYGAKEKGARVVIAN 400

VvSDH1 399 RTYARARELADAVGGDALSLADLNNFHPENGMILANTTSIGMQPKVDETP 448

||:.||:||||.:||||||||||.|||||:||||||||||||||||||||

PgSDH1 401 RTFERAKELADTIGGDALSLADLENFHPEDGMILANTTSIGMQPKVDETP 450

VvSDH1 449 ISKHALKYYSLVFDAIYTPKITRLLREAQESGATIVTGLEMFIGQAYEQF 498

:.|.|||:|:|||||:||||||||||||:|.|||.|:|:||||||||.|:

PgSDH1 451 VPKQALKHYALVFDAVYTPKITRLLREAEECGATTVSGIEMFIGQAYGQY 500

VvSDH1 499 ERFTGLPAPKELFKQFIS 516

||:||||||||||::.:|

PgSDH1 501 ERYTGLPAPKELFRKLMS 518
