# Supplementary material for: Elucidating the role of shikimate dehydrogenase in controlling the production of anthocyanins and hydrolysable tannins in the outer peels of pomegranate
Source: BMC Plant Biol. 2019 Nov 6;19:476. doi: 10.1186/s12870-019-2042-1 (PMC6836501; doi:10.1186/s12870-019-2042-1)
Supplement: Supplementary file 3 — Additional file 3: Figure S1. Phenotype of calli formed from the outer peels of accession UG28 grown on MS plates supplemented with different concentrations of sucrose under light (upper panel) and dark (lower panel) conditions. Figure S2. Fresh weight (gr) of pomegranate peel calli grown for 75 days (collected at five time points) on MS plates supplemented with different sucrose concentrations (1, 2.5, 5, 7.5%) under light and dark conditions. The media were replaced every 15 days. The values presented are the average ± SD of four biological replicates. Figure S3. The fresh weight, dry weight and water content in calli formed from the outer peels of accession UG28 grown on MS plates supplemented with different concentrations of sucrose. The plates were placed in the light (upper panel) and the dark (lower panel), and measurements were taken after 75 days. The data presented represent the mean ± SD of four samples taken from four biological replicates. Statistically significant changes (P < 0.05, using two-way ANOVA) are identified by different letters. Figure S4. Anthocyanin (AT) accumulation in calli formed from the outer peels of accession UG28 grown in the light on MS plates supplemented with different concentrations of sucrose. The peak area of each metabolite was measured using HPLC-DAD and the five ATs were detected. The levels of the mono- and di-glucosides of cyanidin (C-3-G and C-3,5-G), mono- and di-glucosides of pelargonidin (P-3-G and P-3,5-G), and mono-glucoside of delphinidin were measured based on standards of C-3-G and C-3,5-G s. The data presented represent the mean ± SD of four biological replicates. Statistically significant changes (P < 0.05, using two-way ANOVA) are identified by different letters. Figure S5. The content of three aromatic amino acids in calli formed from the outer peels of accession UG28 grown on MS plates supplemented with different concentrations of sucrose. The plates were placed under light or dark conditions. The data pres [file 12870_2019_2042_MOESM3_ESM.pptx]

## Slide 1
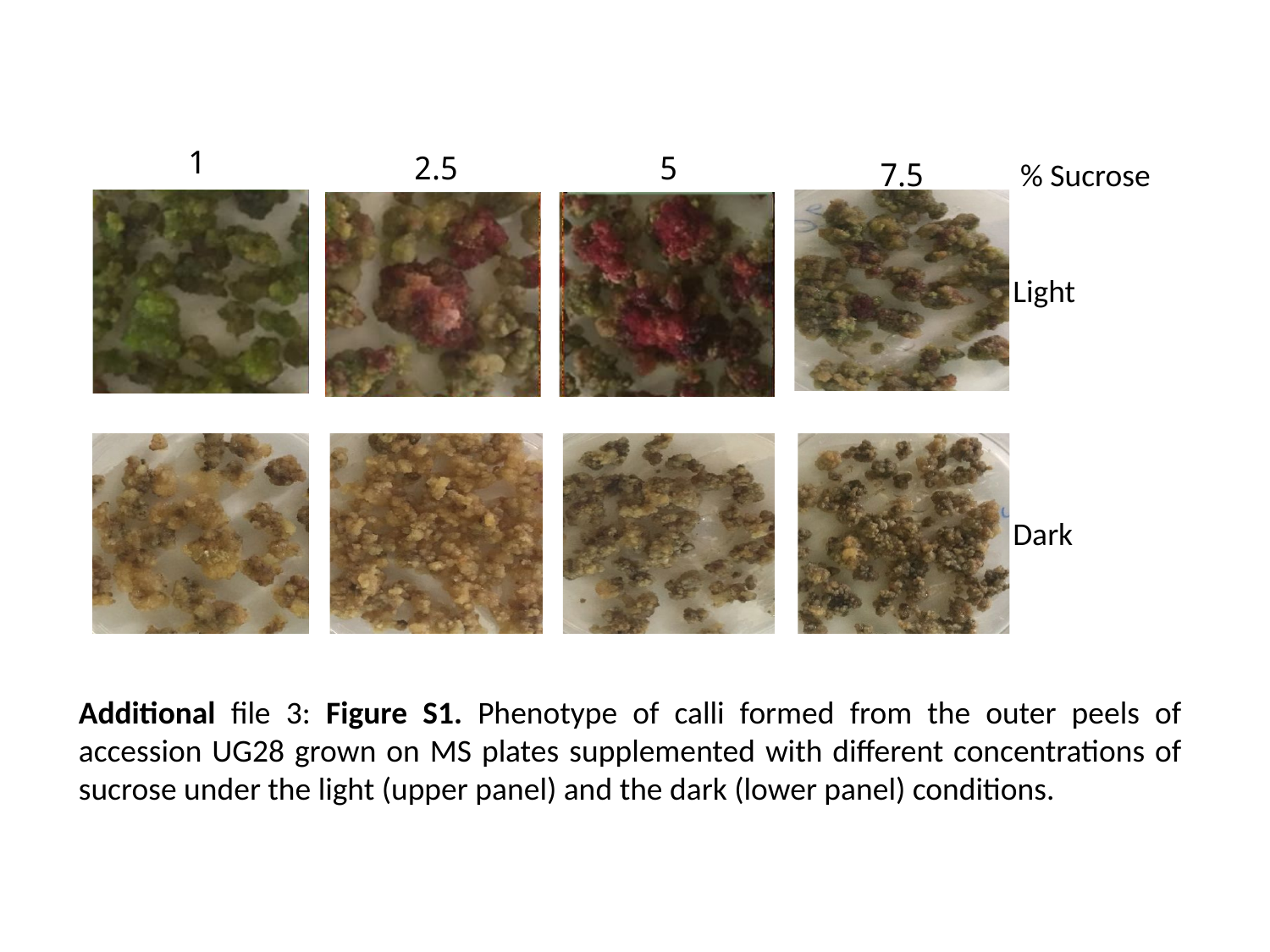

1
2.5
5
7.5
 % Sucrose
Light
Dark
Additional file 3: Figure S1. Phenotype of calli formed from the outer peels of accession UG28 grown on MS plates supplemented with different concentrations of sucrose under the light (upper panel) and the dark (lower panel) conditions.

## Slide 2
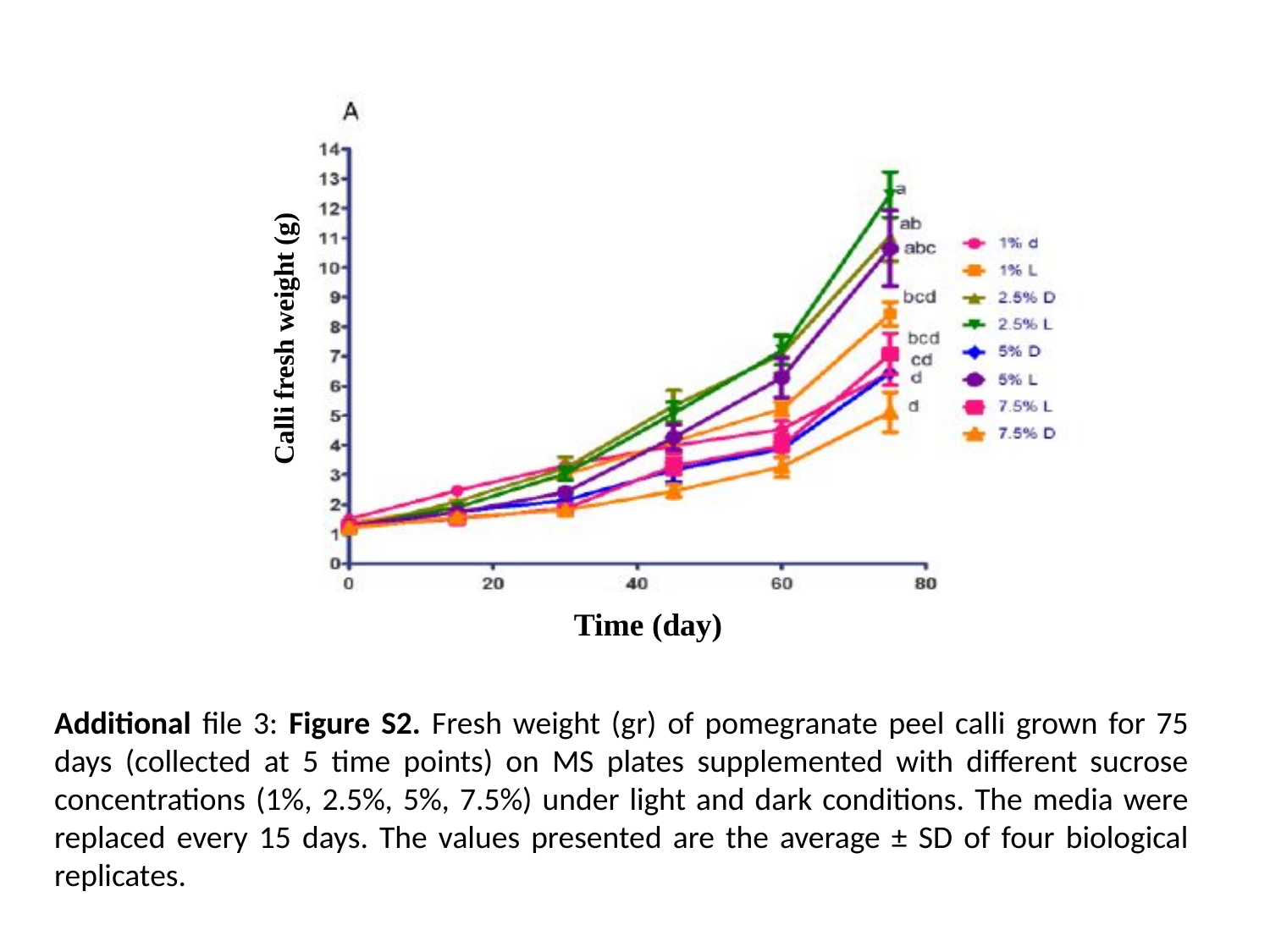

Calli fresh weight (g)
Time (day)
Additional file 3: Figure S2. Fresh weight (gr) of pomegranate peel calli grown for 75 days (collected at 5 time points) on MS plates supplemented with different sucrose concentrations (1%, 2.5%, 5%, 7.5%) under light and dark conditions. The media were replaced every 15 days. The values presented are the average ± SD of four biological replicates.

## Slide 3
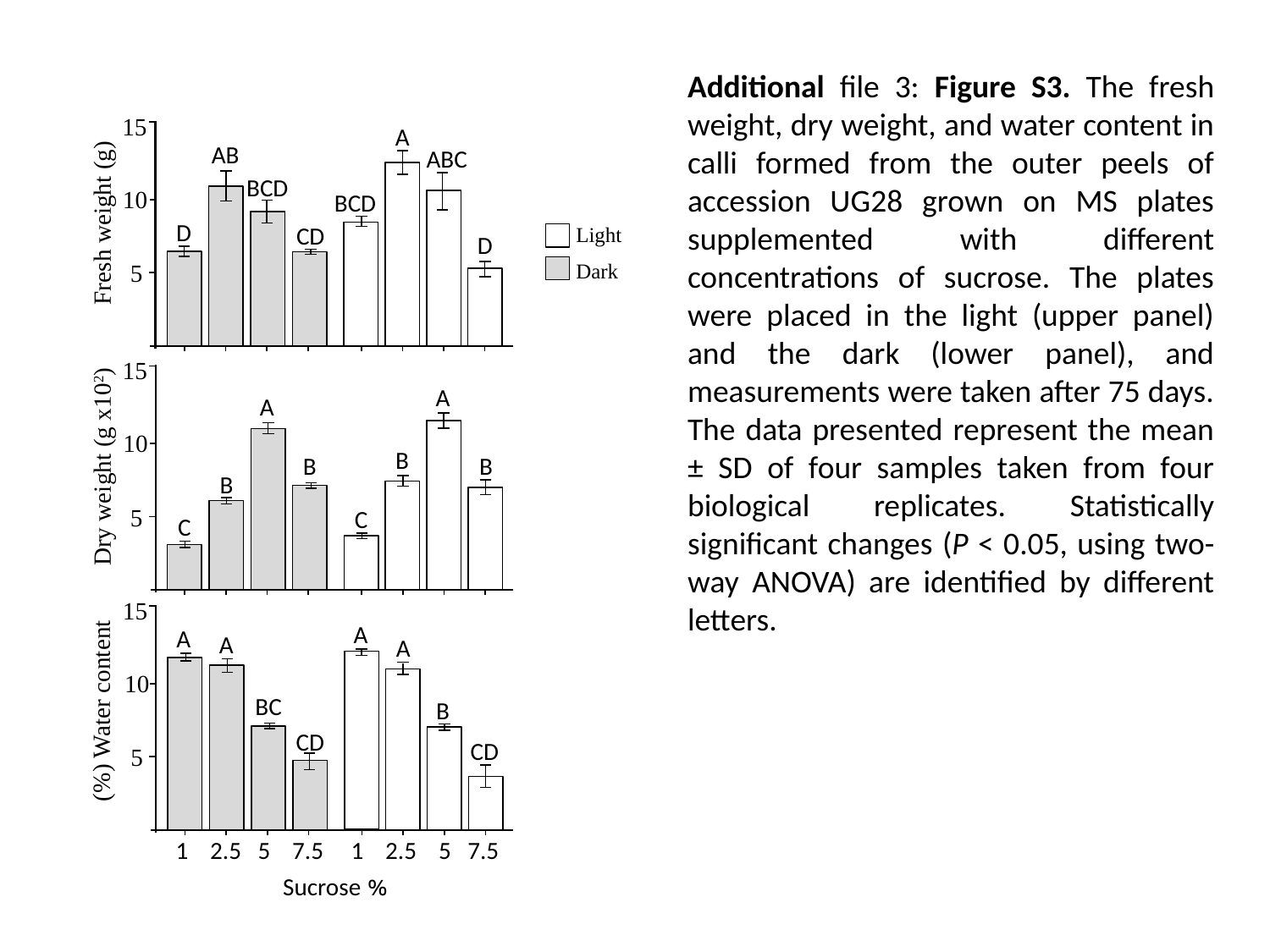

Additional file 3: Figure S3. The fresh weight, dry weight, and water content in calli formed from the outer peels of accession UG28 grown on MS plates supplemented with different concentrations of sucrose. The plates were placed in the light (upper panel) and the dark (lower panel), and measurements were taken after 75 days. The data presented represent the mean ± SD of four samples taken from four biological replicates. Statistically significant changes (P < 0.05, using two-way ANOVA) are identified by different letters.
15
A
AB
ABC
BCD
10
BCD
Fresh weight (g)
D
CD
Light
Dark
D
5
15
A
A
10
B
B
B
Dry weight (g x102)
B
5
C
C
15
A
A
A
A
10
BC
B
Water content (%)
CD
CD
5
1 2.5 5 7.5 1 2.5 5 7.5
% Sucrose

## Slide 4
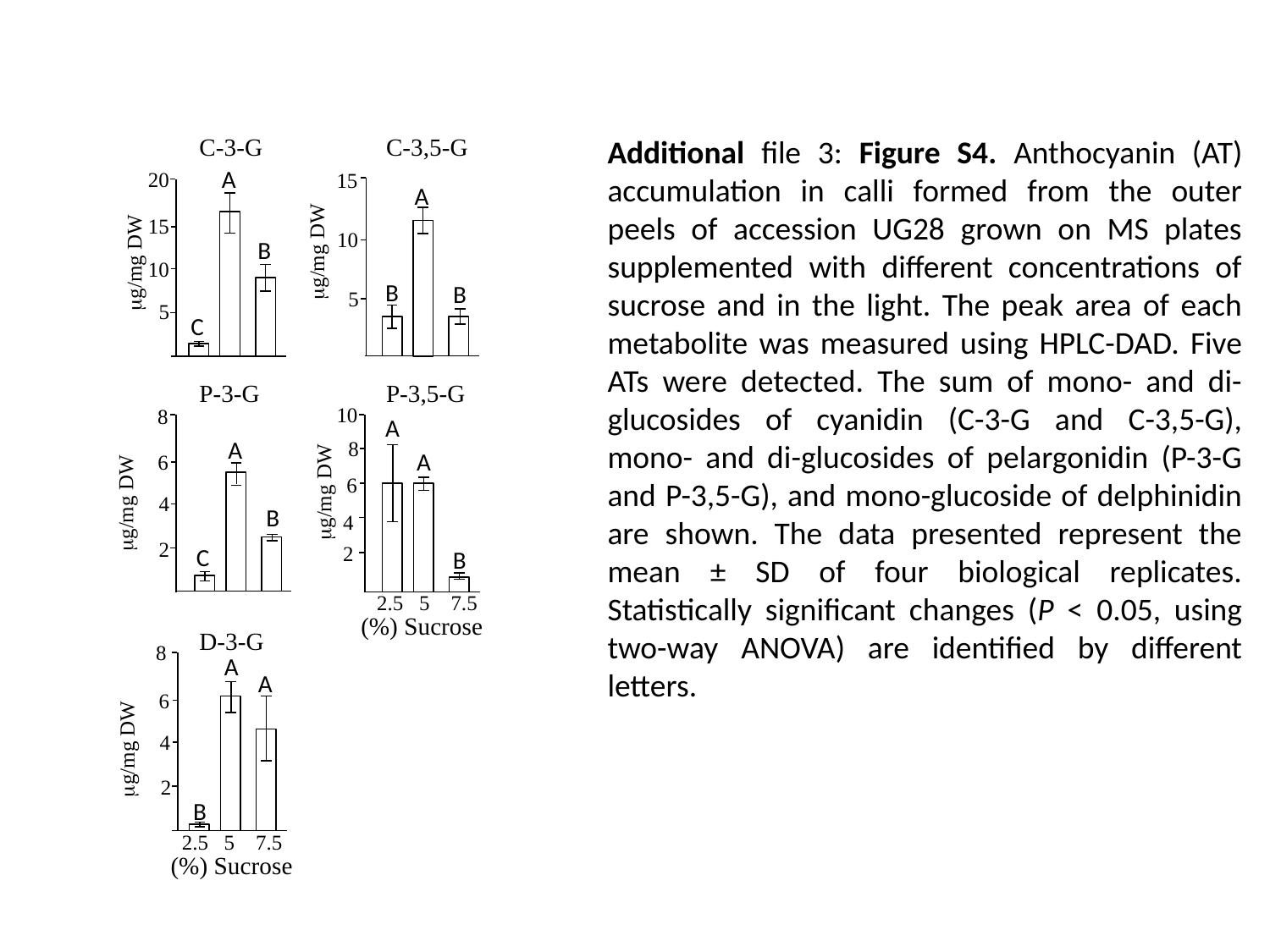

C-3-G
C-3,5-G
A
20
15
A
B
B
15
10
B
μg/mg DW
10
5
5
C
P-3-G
P-3,5-G
10
8
A
A
B
A
B
C
8
6
6
4
4
2
2
2.5 5 7.5
Sucrose (%)
D-3-G
8
A
A
6
4
2
B
2.5 5 7.5
Sucrose (%)
Additional file 3: Figure S4. Anthocyanin (AT) accumulation in calli formed from the outer peels of accession UG28 grown on MS plates supplemented with different concentrations of sucrose and in the light. The peak area of each metabolite was measured using HPLC-DAD. Five ATs were detected. The sum of mono- and di-glucosides of cyanidin (C-3-G and C-3,5-G), mono- and di-glucosides of pelargonidin (P-3-G and P-3,5-G), and mono-glucoside of delphinidin are shown. The data presented represent the mean ± SD of four biological replicates. Statistically significant changes (P < 0.05, using two-way ANOVA) are identified by different letters.
μg/mg DW
μg/mg DW
μg/mg DW
μg/mg DW

## Slide 5
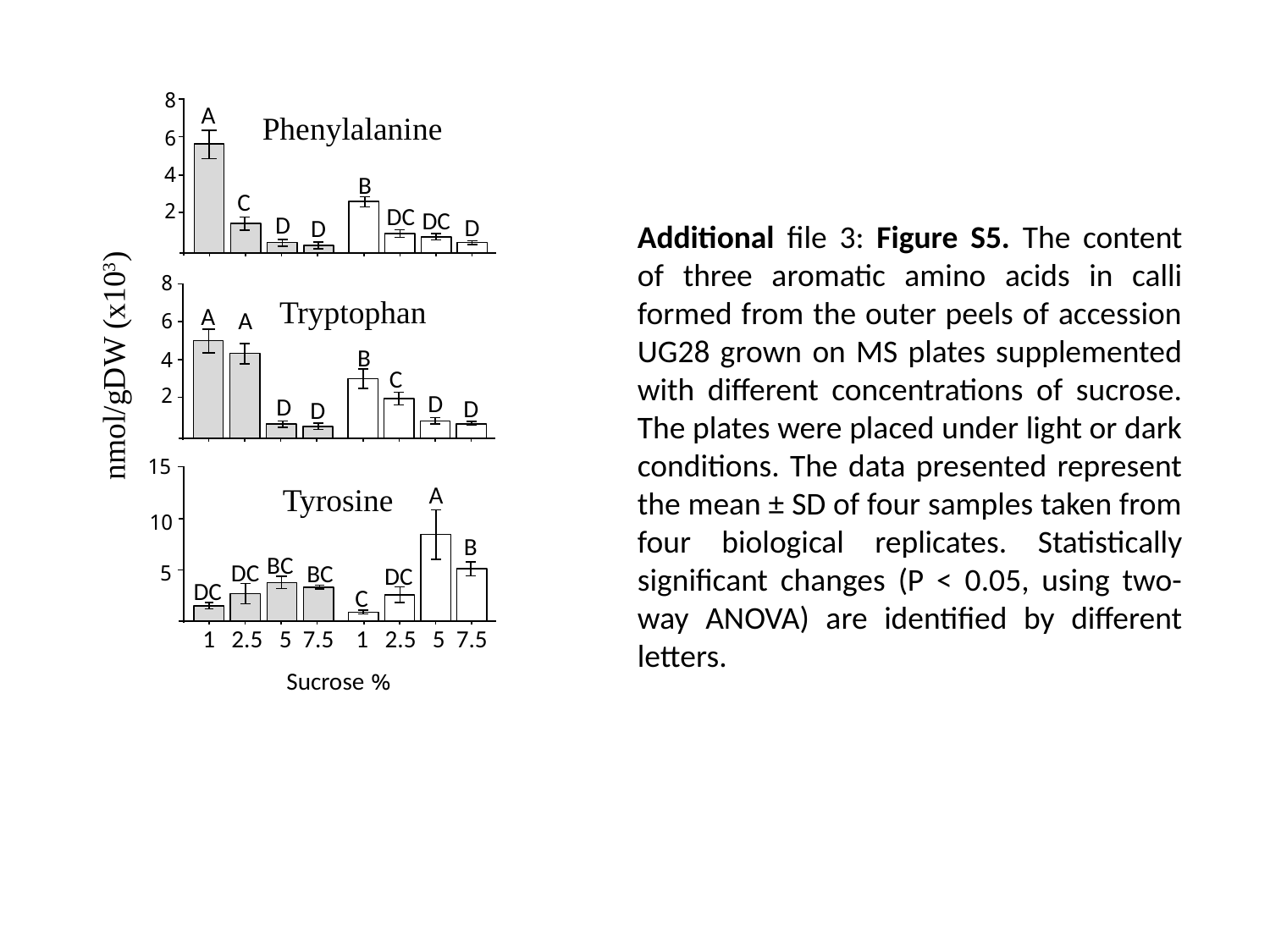

8
A
6
4
B
C
2
DC
DC
D
D
D
Phenylalanine
8
Tryptophan
A
A
6
B
4
nmol/gDW (x103)
C
2
D
D
D
D
15
A
10
B
BC
DC
BC
5
DC
DC
C
Tyrosine
Additional file 3: Figure S5. The content of three aromatic amino acids in calli formed from the outer peels of accession UG28 grown on MS plates supplemented with different concentrations of sucrose. The plates were placed under light or dark conditions. The data presented represent the mean ± SD of four samples taken from four biological replicates. Statistically significant changes (P < 0.05, using two-way ANOVA) are identified by different letters.
1 2.5 5 7.5 1 2.5 5 7.5
% Sucrose

## Slide 6
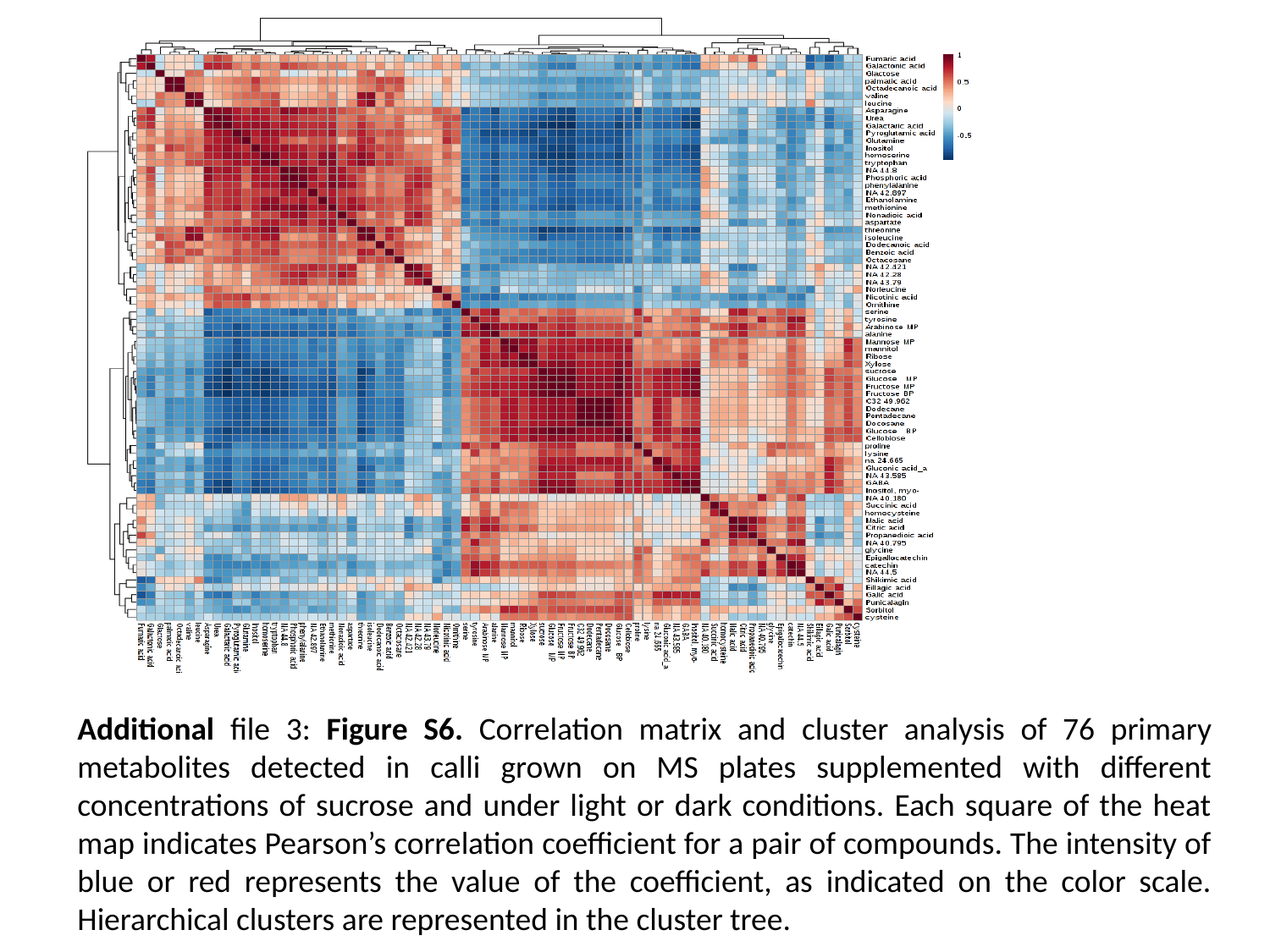

Additional file 3: Figure S6. Correlation matrix and cluster analysis of 76 primary metabolites detected in calli grown on MS plates supplemented with different concentrations of sucrose and under light or dark conditions. Each square of the heat map indicates Pearson’s correlation coefficient for a pair of compounds. The intensity of blue or red represents the value of the coefficient, as indicated on the color scale. Hierarchical clusters are represented in the cluster tree.

## Slide 7
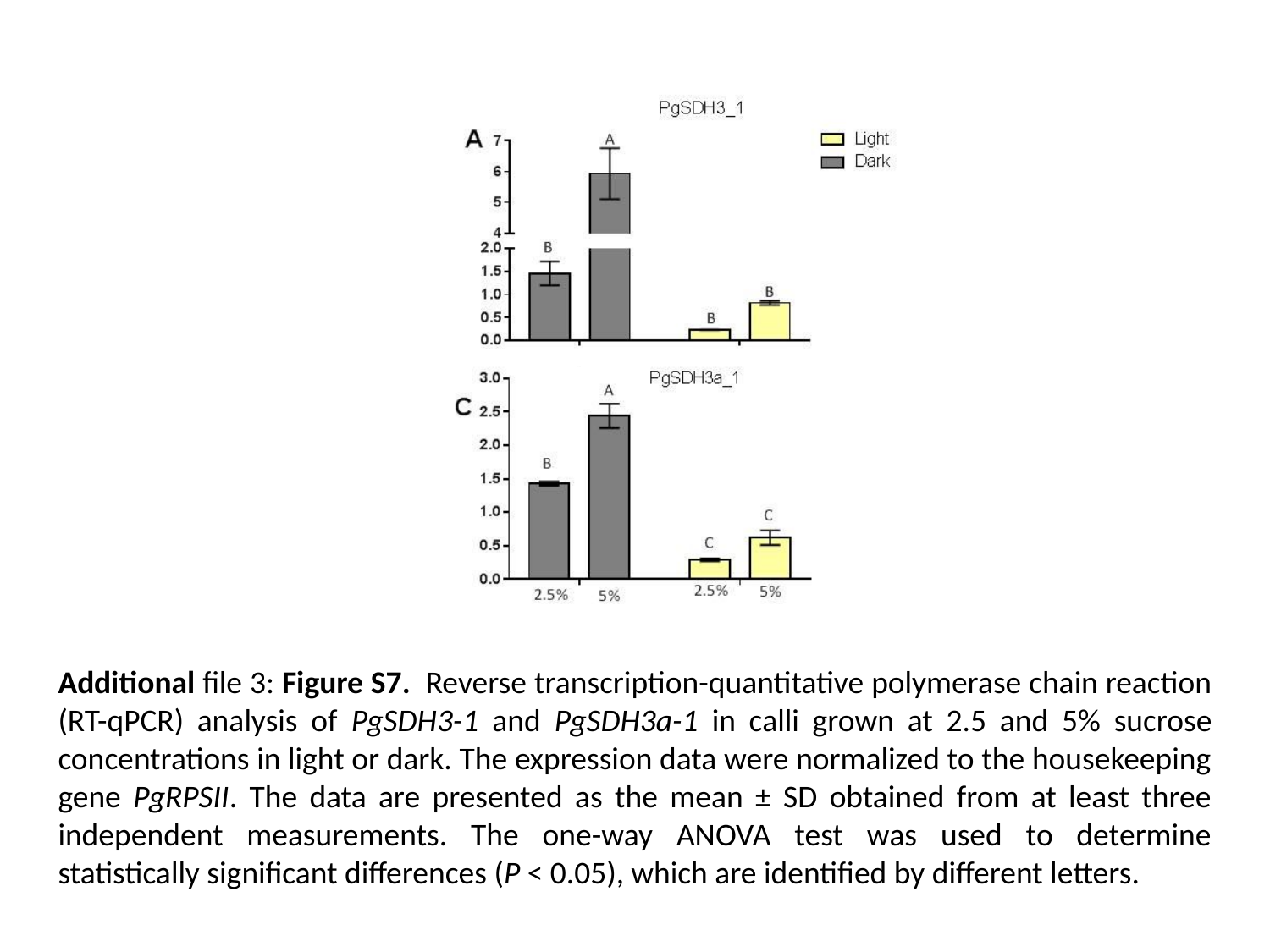

Additional file 3: Figure S7. Reverse transcription-quantitative polymerase chain reaction (RT-qPCR) analysis of PgSDH3-1 and PgSDH3a-1 in calli grown at 2.5 and 5% sucrose concentrations in light or dark. The expression data were normalized to the housekeeping gene PgRPSII. The data are presented as the mean ± SD obtained from at least three independent measurements. The one-way ANOVA test was used to determine statistically significant differences (P < 0.05), which are identified by different letters.
